# Copper-binding anticancer peptides from the piscidin family: an expanded mechanism that encompasses physical and chemical bilayer disruption

**DOI:** 10.1038/s41598-021-91670-w

**Published:** 2021-06-16

**Authors:** Fatih Comert, Frank Heinrich, Ananda Chowdhury, Mason Schoeneck, Caitlin Darling, Kyle W. Anderson, M. Daben J. Libardo, Alfredo M. Angeles-Boza, Vitalii Silin, Myriam L. Cotten, Mihaela Mihailescu

**Affiliations:** 1 Institute for Bioscience and Biotechnology Research, Rockville, MD 20850 USA; 2 Department of Physics, Carnegie Mellon University, Pittsburgh, PA 15213 USA; 3 Center for Neutron Research, National Institute of Standards and Technology, Gaithersburg, MD 20899 USA; 4 University of Rochester School of Medicine and Dentistry, Rochester, NY 14620 USA; 5 Department of Biological Sciences, Clemson University, Clemson, SC 29634 USA; 6 Biomolecular Measurement Division, National Institute of Standards and Technology, Gaithersburg, MD 20899 USA; 7 Department of Chemistry and Institute of Materials Science, University of Connecticut, Storrs, CT 06269 USA; 8 Department of Applied Science, William and Mary, Williamsburg, VA 23185 USA

**Keywords:** Membrane structure and assembly, Chemical modification, Biochemistry, Biophysics, Structural biology, Chemistry

## Abstract

In the search for novel broad-spectrum therapeutics to fight chronic infections, inflammation, and cancer, host defense peptides (HDPs) have garnered increasing interest. Characterizing their biologically-active conformations and minimum motifs for function represents a requisite step to developing them into efficacious and safe therapeutics. Here, we demonstrate that metallating HDPs with Cu^2+^ is an effective chemical strategy to improve their cytotoxicity on cancer cells. Mechanistically, we find that prepared as Cu^2+^-complexes, the peptides not only physically but also chemically damage lipid membranes. Our testing ground features piscidins 1 and 3 (P1/3), two amphipathic, histidine-rich, membrane-interacting, and cell-penetrating HDPs that are α-helical bound to membranes. To investigate their membrane location, permeabilization effects, and lipid-oxidation capability, we employ neutron reflectometry, impedance spectroscopy, neutron diffraction, and UV spectroscopy. While P1-apo is more potent than P3-apo, metallation boosts their cytotoxicities by up to two- and seven-fold, respectively. Remarkably, P3-Cu^2+^ is particularly effective at inserting in bilayers, causing water crevices in the hydrocarbon region and placing Cu^2+^ near the double bonds of the acyl chains, as needed to oxidize them. This study points at a new paradigm where complexing HDPs with Cu^2+^ to expand their mechanistic reach could be explored to design more potent peptide-based anticancer therapeutics.

## Introduction

In recent years, global concerns have arisen regarding multi-drug resistant (MDR) pathogens and cancers^1–7^. Importantly, chronic bacterial infections represent prevalent comorbidity in cancer patients^8–13^. They also lead to chronic inflammation that can result in cancer^14^. Given the intersection between life-threatening infections and cancers, there is a strong motivation to develop therapeutics with broad-spectrum activity to treat both conditions simultaneously. With this regard, several membrane-interacting host-defense peptides (HDPs) with cytotoxic properties against cancer cells have emerged as promising templates^7,8,15–22^. However, a major challenge to developing them into efficacious, safe, and affordable therapeutics is the lack of structure-action relationship (SAR) studies on beneficial peptides, as needed to characterize their biologically-active conformations and minimum motifs required for function. Overall, there is also a strong need to identify chemical modifications that boost peptide potencies, and thereby reduce dosage, side effects, and cost^7,19,22–30^.

In this study, we investigate piscidins, which are cationic histidine-rich membrane-interacting HDPs widely expressed in teleost fish^31–33^. Through a combination of SAR studies, we demonstrate that coordinating these amphipathic peptides with Cu^2+^ is an effective chemical strategy to enhance their cytotoxicity on cancer cells. On a mechanistic level, we show that these biological effects correlate with the ability of the peptides to not only physically but also chemically impair lipid membranes.

As evolutionary-optimized molecules from the innate immune system, cationic HDPs favorably interact with anionic danger signals on the surface of pathogenic bacterial and cancer cells^3,7,8,15,19,24,34–36^. While most membrane-interacting HDPs target pathogens, several of them are cytotoxic to cancer cells, resulting in their classification as anticancer peptides (ACPs)^7,8,15,19–21^. Significant advantages of ACPs include their high specificity and low incidence of resistance due to the short timescale of their interactions with vital cell membranes, good biocompatibility due to low toxicity and side effects, and dual ability to kill neoplastic (abnormal) cells and exert anti-inflammatory function^7,8,15,19,21,24,25,27,29,30,37^. Anionic molecules more represented on cancer than healthy cells include phosphatidylserine (PS), and anionic carbohydrates on glycosylated lipids and proteins^7,8,15,19,24,38^. Other determinants that may impart specificity to ACPs include the altered cholesterol content, low pH, and increased membrane potential and surface area associated with neoplastic cells^7,15^.

Following their initial interactions with cell surfaces, ACPs induce cell death via necrotic and/or apoptotic pathways depending on their concentration and the specific cells that are targeted^7,8,15,17,19,24,36,39^. In the case of necrosis, cells display blebbing of their plasma membranes, suggesting that cell death is due to membrane lysis. Specific mechanisms of action for membrane disruption have been proposed, including membrane permeabilization or rupture via the formation of toroidal pores or carpets^7,15,19,36^. In terms of apoptosis, some ACPs get internalized inside the cells and interact with intracellular targets, ultimately triggering programmed cell death. Intracellular effects may include the disruption of mitochondrial membranes, which have a large membrane potential and anionic lipid content (e.g. cardiolipin, CL), and thus attract cationic ACPs^15,17,19,40,41^. In particular, high mitochondrial reactive oxygen species (ROS) levels have been associated with agents that induce apoptotic cell death^17,40–43^.

Marine animals produce HDPs that are highly potent against a wide range of harmful cells and diseases, including cancer^21,44–46^. Particularly promising peptides include the piscidins from hybrid striped bass^31–33,47^ and tilapia^48^, and their homologs in other fish species: pleurocidin from flounder^49^, epinecidin-1 from grouper^50^, and chrysophsin from red sea bream^51^. Piscidins are characterized by their abundance of histidine residues^31^. The isoforms piscidin 1 (P1) and piscidin 3 (P3) were co-discovered in the mast cells of hybrid striped bass^47^. Both peptides are highly potent against both Gram-positive and Gram-negative bacteria, with minimum inhibitory concentrations (MIC) in the range of 1 to 20 μmol/L^33,47,52,53^.

While P1 and P3 are 68% homologous and have almost identical α-helical structures in a lipid bilayer environment^53–58^, P1 is a more potent antimicrobial agent^47,53,59^. It is active against viruses such as HIV-1^60^, coronaviruses^61^, and pseudorabies^62^. Its cytotoxic properties against several cancer cell lines were reported by Lin et al. (2012)^39^. The authors found that P1 induced apoptosis in HT1080 cells at low concentrations while it had necrotic effects at high concentrations.

In previous biophysical studies of P1/3, we demonstrated that the stronger membrane permeabilizing effects and pH-resiliency of P1 correlate with its higher histidine content and a membrane-inserted state that places its C-terminus in the bilayer core^56^. In contrast, P3 uses its N-terminus to direct membrane insertion. Recently, we showed that both P1 and P3 bind Cu^2+^/Ni^2+^ ions using an amino-terminal copper/nickel (ATCUN) binding motif^59,63–65^. The ATCUN motif (XXH), which is highly conserved in the piscidin family, exists in other proteins such as albumins^66–69^. The metal is coordinated by four nitrogens, including the nitrogen from the N-terminus, the two subsequent backbone amide nitrogens, and the δ-nitrogen of His-3 arranged in a distorted square planar coordination geometry^68,70,71^. The ATCUN motif requires the deprotonation of the three backbone nitrogen sites, and thus metallation is more favorable at basic pH^59,63,64^. Once bound to Cu^2+^, ATCUN-containing peptides become distinctively pink, a useful property to ascertain their metallation state under changing sample conditions^63,64^. Given the ability of Cu^2+^ to form ROS, synthetic peptides containing the ATCUN motif have been designed to generate radicals and damage harmful cells and pathogens, including in vivo^67,69,71–77^. Of particular interest here is the pioneering work of Pauling and colleagues who demonstrated the ability of the GGH-Cu^2+^ peptide to kill cancer cells through a mechanism involving the release of the metal ion when the complex reaches the acidic microenvironment of tumor cells^76^. Free Cu^2+^ then reacts with metabolites in the cancer cells to form ROS and damage vital cellular components (e.g., DNA).

As cell penetrating peptides with built-in host defense functions, P1 and P3 can specifically carry Cu^2**+**^ to and across bacterial cell membranes, enabling the metal ion to form ROS that oxidize unsaturated fatty acids in lipid membranes and nick intracellular DNA^59,73,75^. Recent studies of Cu^2+^-bound P1 showed that the presence of oxidized lipids in model membranes improves its membrane permeabilization effects, probably because the oxidized lipids weaken the integrity of the membrane barrier and the loss of a positive charge by the peptide upon metal-ion binding facilitates membrane insertion^63^.

As demonstrated here, Cu^2+^-coordination to P1 and P3 before they are added to cancer cells improves their cytotoxicities on cancer cells, with P3 responding to metallation much more dramatically than P1. In investigating this remarkable result on a mechanistic level, we establish a correlation between the enhanced cytotoxicity, deeper membrane insertion, and lipid peroxidation capability of P3-Cu^2+^ compared to P3. Thus, this study identifies the metallation of HDPs with Cu^2+^ as an effective strategy to improve their anticancer properties via the expansion of their mechanistic reach, so that both physical and chemical disruptive effects of membranes are elicited.

## Results

### Effect of Cu^2+^-metallation on the cytotoxicity of P1 and P3 against several cancer cell lines

**Figure 1:**
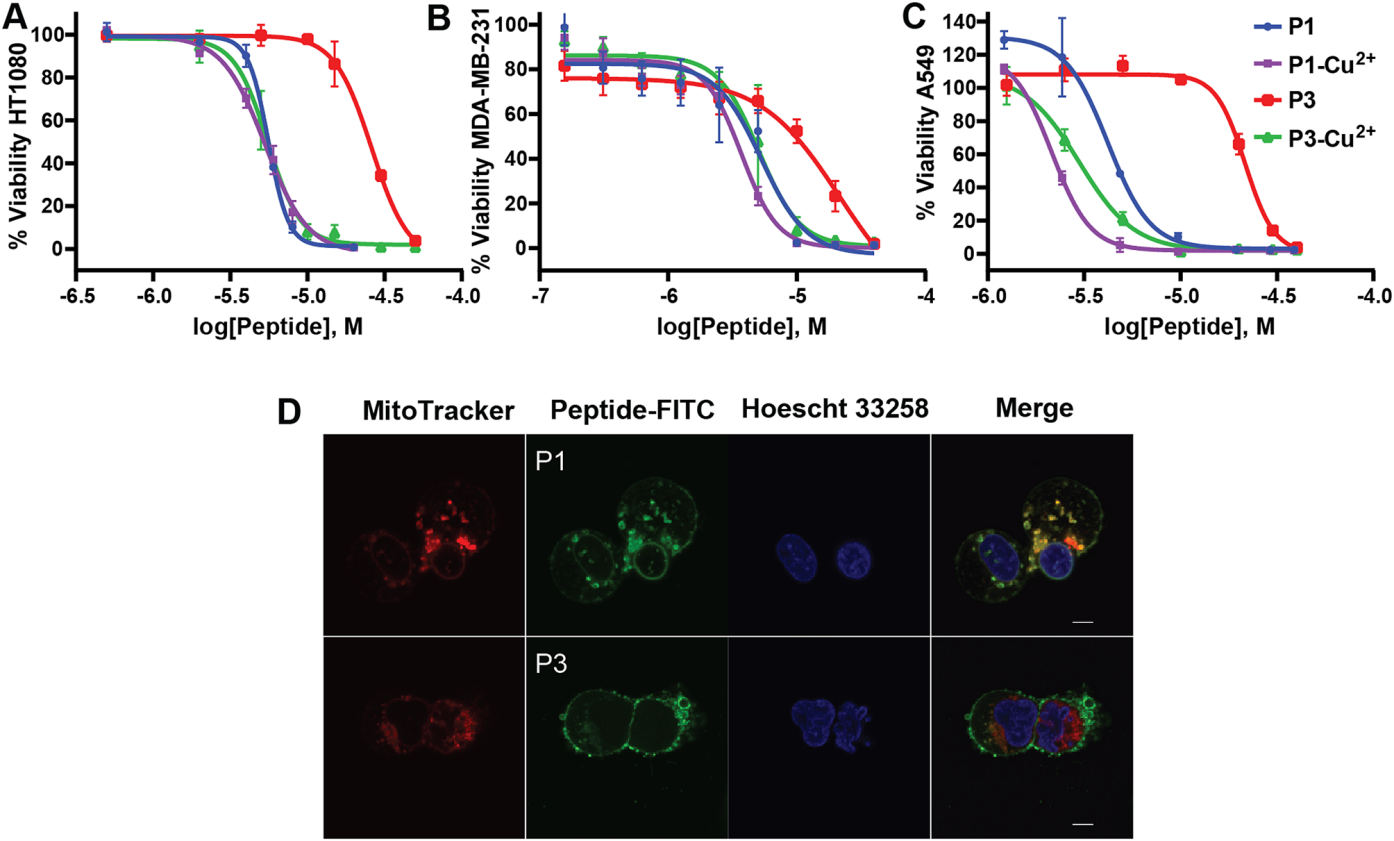
Cytotoxicity assays for the apo- and holo-states of P1 and P3 acting on cancer cells. Fibrosarcoma (HT1080), breast (MDA-MB-231), and lung (A549) cancer cell lines (panels A, B, and C, respectively) were used to measure cell viability after 24 h of treatment with different concentrations (M = mol/L) of P1 and P3 in the apo- and Cu^2+^-bound states. Error bars are standard deviations based on triplicates. The IC_50_ values are summarized in Table 1. Similar results were obtained after 48 h (Fig. S1). The peptides were metallated using a 1:1 stoichiometric amount of CuCl_2_. Panel C: MD-MBA-231 cells exposed to 100 nM MitoTracker (red), 5 µg/mL Hoescht 33,258, and 4 µmol/L FITC-labeled P1 or P3 (green) for 20 min. Top row: FITC-P1 stains the cellular membrane and nuclear envelope and co-localizes with MitoTracker. Bottom row: FITC-P3 stains the cellular membrane and there is co-localization with MitoTracker (left cell). Scale bar represents 5 µm.

Prior studies by Lin et al. (2012) demonstrated that P1 induces apoptosis and necrosis in HT1080 (fibrosarcoma) cancer cells^39^. The authors also characterized the activity of P1 on A549 (lung epithelial) and HeLa (cervical) cancer cells. However, P3 was not tested. Here, we investigated the activities of P1 and P3 in the apo-state and holo (Cu^2+^-bound) form on these three cell lines, as well as the MDA-MB-231 (breast) cancer cells. As shown in Figs. 1 and S1A, and Table 1, and in agreement with the work of Lin et al., P1 has considerable cytotoxic effects against all cancer cell lines tested. Based solely on the cell viability profiles for P1 and P3 in the investigated concentration range (0–50 µmol/L), apo-P3 is much less cytotoxic than apo-P1. Remarkably, the Cu^2+^-bound form of P3 exhibits a significant boost in killing ability. For instance, based on Fig. 1 and the average IC_50_ values listed in Table 1, metallation with Cu^2+^ increases the efficacy of P3 by a factor of ≈7 on the A549 and ≈5 on the HT1080 cells. In the case of P1, Cu^2+^-binding improves activity by up to a factor of ≈2 (e.g., A549, MDA-MB-231). Free Cu^2+^ tested at up to 30 µmol/L was not cytotoxic under the same conditions (Fig. S1B). Thus, while it is not known whether the peptides remain bound to Cu^2+^ throughout the process of killing cancer cells, it is essential for Cu^2+^ and the peptides to be bound to each other in order to reach low IC_50_ values. The Cu^2+^-bound peptides did not exhibit this strong level of cytotoxicity on normal MRC-5 (lung) fibroblast cells (Fig. S1C), in agreement with the data previously obtained for apo-P1 on MRC-5, HGF-1 (gingival) and OMF (mucosal) fibroblast cells^39,78^. Higher selectivity of the cationic peptides for cancer cells is very likely related to different chemical conditions of their membrane environment, as explained in the introduction.

**Table 1:** IC_50_ for P1 and P3 acting on cancer cells in the apo- and holo-forms.

IC_50_ (µmol/L)	HT1080	MDA-MB-231	A549	HeLa
P1	5.58^a^ (5.41–5.77)	5.83^b^ (4.82–7.04)	4.20^a^ (3.71–4.65)	2.22^a^ (1.37–3.61)
P1-Cu^2+^	5.31 (4.91–5.75)	3.92^b^ (3.54–4.33)	2.15 (1.94–2.30)	2.17 (1.32–3.60)
P3	26.01 (23.7–28.6)	17.5^b^ (11.8–26.0)	21.6 (20.4–23.7)	18.1^b^ (10.8–30.4)
P3-Cu^2+^	5.52 (5.03**–**6.05)	5.25^b^ (4.32–6.37)	2.99 (2.60–3.34)	1.97 (1.24–3.12)

The range of concentrations corresponding to the 95% confidence interval are provided in parentheses.

^a^From this work. Comparison to data in Lin et al.^39^ is provided in the text.

^b^First data point at 0.16 µmol/L was not used to extract IC_50_ values due to large deviation from the trend line but all data points are displayed in Fig. 1.

We note that Lin et al.^39^ obtained slightly higher IC_50_ values on the HT1080, A549, and HeLa cells. In comparing their experimental protocol to ours, the most significant difference appears to be the use of different approaches to assess the concentrations of the peptide solutions. Piscidins 1 and 3 lack strong chromophores, preventing the common determination of molar concentration from the measurement of UV absorbance at 280 nm. As a result, we relied on amino acid analysis to derive molarity. In contrast, Lin et al.^39^ used peptide mass. Since highly cationic peptides have an undetermined number of counterions, the total sample weight will tend to overestimate both the net amount of peptide and IC_50_ values.

Microscopy images using P1 and P3 labeled with a green fluorescein isothiocyanate (FITC) label attached to their N-terminus show that they have a preference for membranous organelles (see Fig. 1 for MD-MBA-231 cells and Fig. S2 for HeLa cells). After 20 min of incubation with 4 μmol/L of the FITC-labeled peptides, the cells undergo morphological changes. In both MD-MBA-231 and HeLa cells, FITC-P1 co-localizes with the plasma membrane, the nuclear envelope, and red MitoTracker. FITC-P3 interacts with the plasma membrane of the MDMBA-231 as well as with the MitoTracker. Added to the HeLa cells, it also co-localizes with the nuclear membranes. We previously showed that P1 and P3 have strong affinities for membranes and P1 is more membrane active than P3^53,56–58,79^. It is thus plausible that the stronger cytotoxicity of P1 compared to P3 relates to its stronger membrane activity observed on model and bacterial cell membranes.

**Figure 2:**
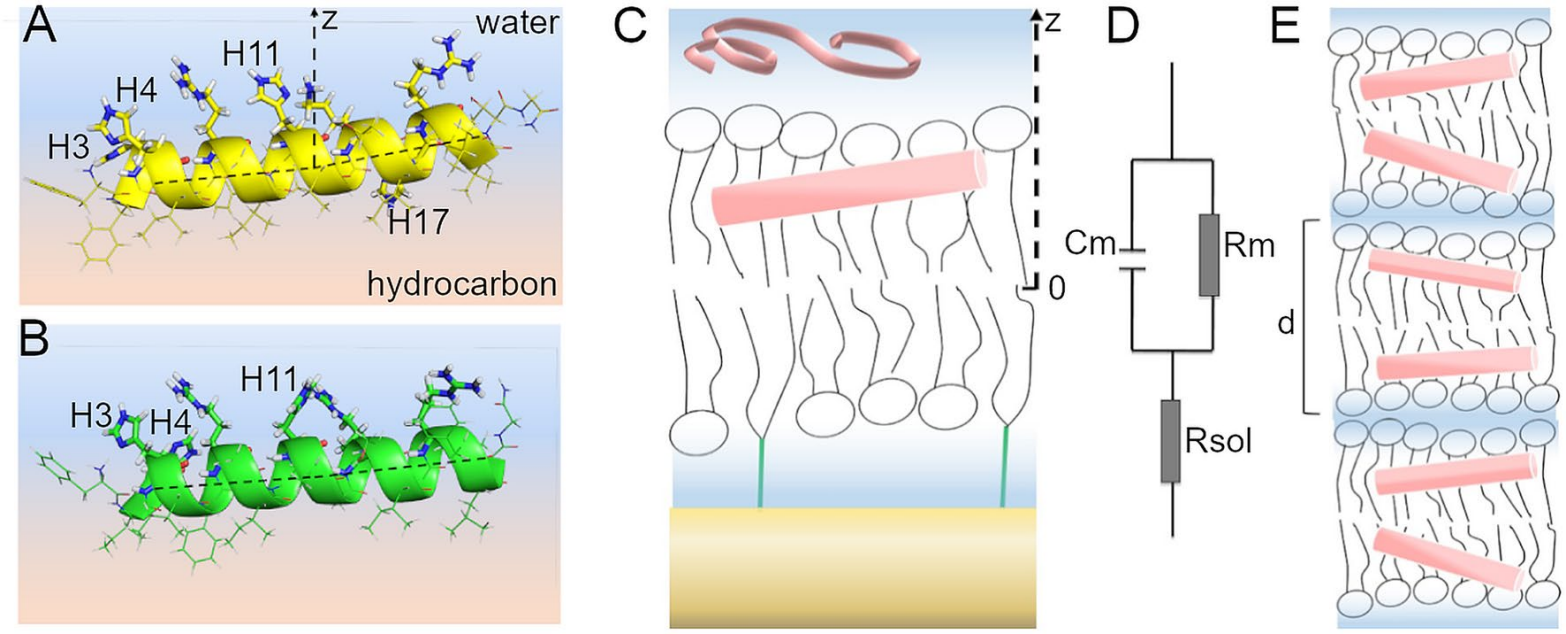
Techniques for investigating peptide-bilayer structural interactions. (**A**) High-resolution structure of piscidin P1: FFHHIFRGIVHVGKTIHRLVTG (MW 2571) and (**B**) Piscidin P3: FIHHIFRGIVHAGRSIGRFLTG (MW 2492). The shown structures were determined by solid state NMR in 4:1 POPC/cholesterol bilayers at P/L = 1:40^58^. The corresponding Protein Data Bank IDs are 6PF0 (P1) and 6PEZ (P3). The α-helices of the peptides lay at the bilayer-water interface, adopting orientations that are almost parallel to the bilayer surface. (**C**) Cartoon describing the tethered bilayer membrane used for Surface Plasmon Resonance (SPR), Electrical Impedance Spectroscopy (EIS) and neutron reflectometry (NR). The tethered molecules (green) create a 10 Å thick sub-membrane aqueous space. (**D**) The simplest electric circuit model to describe the surface-supported bilayer, characterized by the membrane capacitance (Cm), resistance (Rm), and solvent resistance (Rsol). (**E**) Oriented lipid multilayers with peptide incorporated as described in the Methods for the neutron diffraction (ND) experiments. The repeat spacing (d) denotes the dimension of the repeat unit (thickness of the bilayer with its hydration layer).

Since the FITC group is attached to the amino terminal group of P1 and P3, FITC-P1/3 could not be metallated. As a result, we were not able to use these fluorescently-labeled peptides to determine how metallation affects cellular localization. However, P1 is highly membrane active and metallation does not affect its IC_50_ by more than a factor of 2 (Table 1), thus binding to Cu^2+^ very likely does not affect its mechanism of action and ability to strongly interact with membranous organelles. By comparison, P3 shows a much larger increase in IC_50_ upon Cu^2+^-binding (Table 1). To gain insights into the structural aspects of the peptide-bilayer interactions that could explain how metallation increases the cytotoxicity of P1 and P3 on cancer cells, we employed several complementary biophysical techniques, as described below (Fig. 2).

### Kinetics of lipid binding and membrane permeation for P1, P3, P1-Cu^2+^, and P3-Cu^2+^

**Figure 3:**
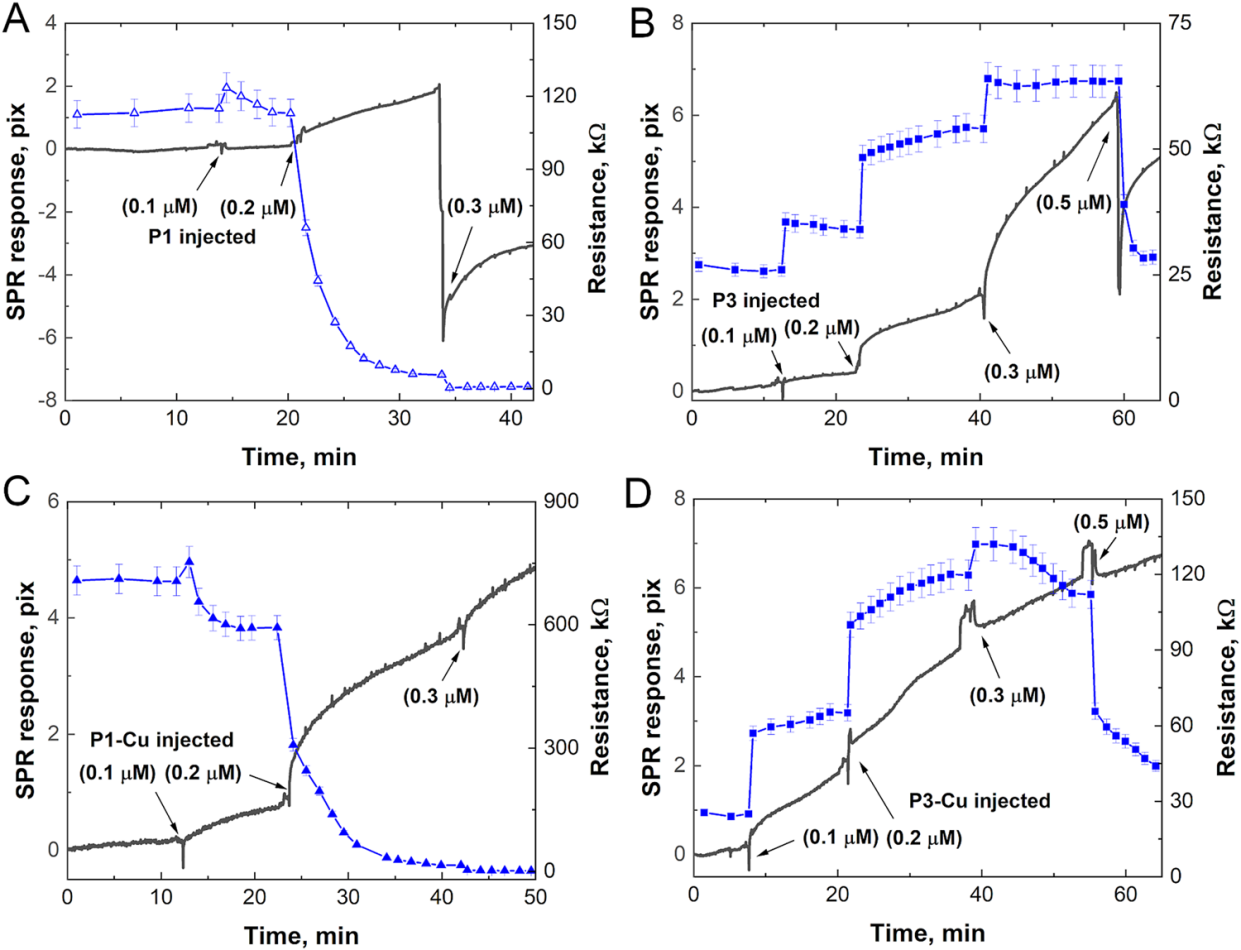
SPR/EIS of P1 and P3 with or without Cu^2+^. The black curves are the SPR responses, and the blue curves are the calculated bilayer resistance values from EIS measurements. Peptides were added in small increments on top of a stable POPC tBLM. SPR/EIS signals were collected simultaneously as a function of time: (**A**) P1, (**B**) P3, (**C**) P1-Cu^2+^, and (**D**) P3-Cu^2+^. The peptides were metallated using a 1:1 stoichiometric amount of CuCl_2_. See also Fig. S3. (µM = µmol/L).

Planar, tethered bilayer membranes (tBLM) supported on gold-coated surfaces (Fig. 2C) were made using 1-palmitoyl-2-oleoyl-*sn*-glycero-3-phosphocholine (POPC) to mimic cancer cell membranes^80,81^. Combined, surface plasmon resonance (SPR)/electrical impedance spectroscopy (EIS) measurements (Fig. 3) were conducted by injecting the peptide in small increments while simultaneously observing (1) the kinetics associated with the peptides binding to the bilayer (SPR) and (2) the formation of peptide-lined defects that increase membrane permeation and conductance (EIS). To capture the threshold needed for the peptides to bind to the bilayer and induce permeation, we started in the very low concentration range (0.1 to 0.5 μmol/L). While this concentration range is well below the IC_50_ values reported for P1 and P3 in Fig. 1, the SPR/EIS technique is sensitive enough to detect bilayer-peptide interactions in the sub-lethal regime. In this low peptide concentration regime, the SPR signal increases slowly as the peptides attack the membrane surface (Fig. 3A, B, black line). Fluctuations (drops) in the SPR signal, seen here at 0.3 μmol/L P1 and 0.5 μmol/L P3, are likely due to a dislocation of ions, lipid, or loosely bound material peptides as peptides accumulate on the surface. The aforementioned fluctuations are not as apparent in the SPR signals when using holo-P1/3 (Fig. 3C, D) compared to apo-P1/3 (Fig. 3A, B), suggesting that the Cu^2+^-bound peptides are more efficient at integrating themselves within the bilayer interior, precluding a significant mass dislocation at the tBLM surface.

As concentration increases further, peptides induce transbilayer defects that create pathways for ion conduction through the otherwise electrically sealing bilayer. This is best captured in the EIS signal (Fig. 3, blue curves). Such membrane defects are seen as a sharp drop in bilayer resistance from 125 to 0 kΩ, at a concentration as low as 0.2 μmol/L for P1 (Fig. 3A). In contrast to P1, P3 shows a step-wise accumulation on the surface of the bilayer as the concentration increases. It is only at 0.5 μmol/L that P3 induces a drop in resistance (Fig. 3B, blue line). Based on the EIS measurements, P1 exhibits a more aggressive behavior, requiring a dose five times lower than for P3 to open the bilayer. Interestingly, the IC_50_ of P1 is five-fold lower than that of P3 in the cytotoxicity assays presented above (Table 1), suggesting that when we compare the two peptides the more potent one is commensurately more membrane active. Overall, the SPR/EIS measurements reveal the differing bilayer interactions and efficacies of these two membrane-active peptide homologs. They also show that the holo-forms of P1/3 are generally more aggressive (larger drop in resistance) than the apo-forms. Tested at higher concentrations (e.g., 3 μmol/L) to match the IC_50_ concentrations listed in Table 1, the holo-forms remain more membrane disruptive than the apo-forms (Fig. S3). Clearly, the presence of the metal ion, carried through the ATCUN motif, modifies the interactions of P1 and P3 with the bilayer, resulting in a more efficient integration and permeabilization of the bilayer core. Next, to correlate these observations with direct structural information on the peptide-bilayer complexes, we employed surface scattering techniques to study planar bilayer systems exposed to the peptides.

### Distributions of P1, P3, P1-Cu^2+^, and P3-Cu^2+^ in tethered lipid bilayers

NR was used here to determine the ensemble and time averaged spatial distribution of the peptides in the lipid bilayer. The NR experiments were set up on a POPC tBLM (Fig. 2C) for a direct comparison with the SPR/EIS studies presented above. Before the addition of a given peptide to the tBLM, NR measurements and density calculations of the tBLM reference system were performed without peptide to ensure the formation of a complete bilayer. Fresh tBLMs were independently prepared for P1, P3, P1-Cu^2+^, and P3-Cu^2+^. These peptides were injected at a fixed concentration of 3 µmol/L in the aqueous compartment above the tBLM and allowed to interact with the bilayer before taking measurements (see Methods and Fig. S4).

**Figure 4:**
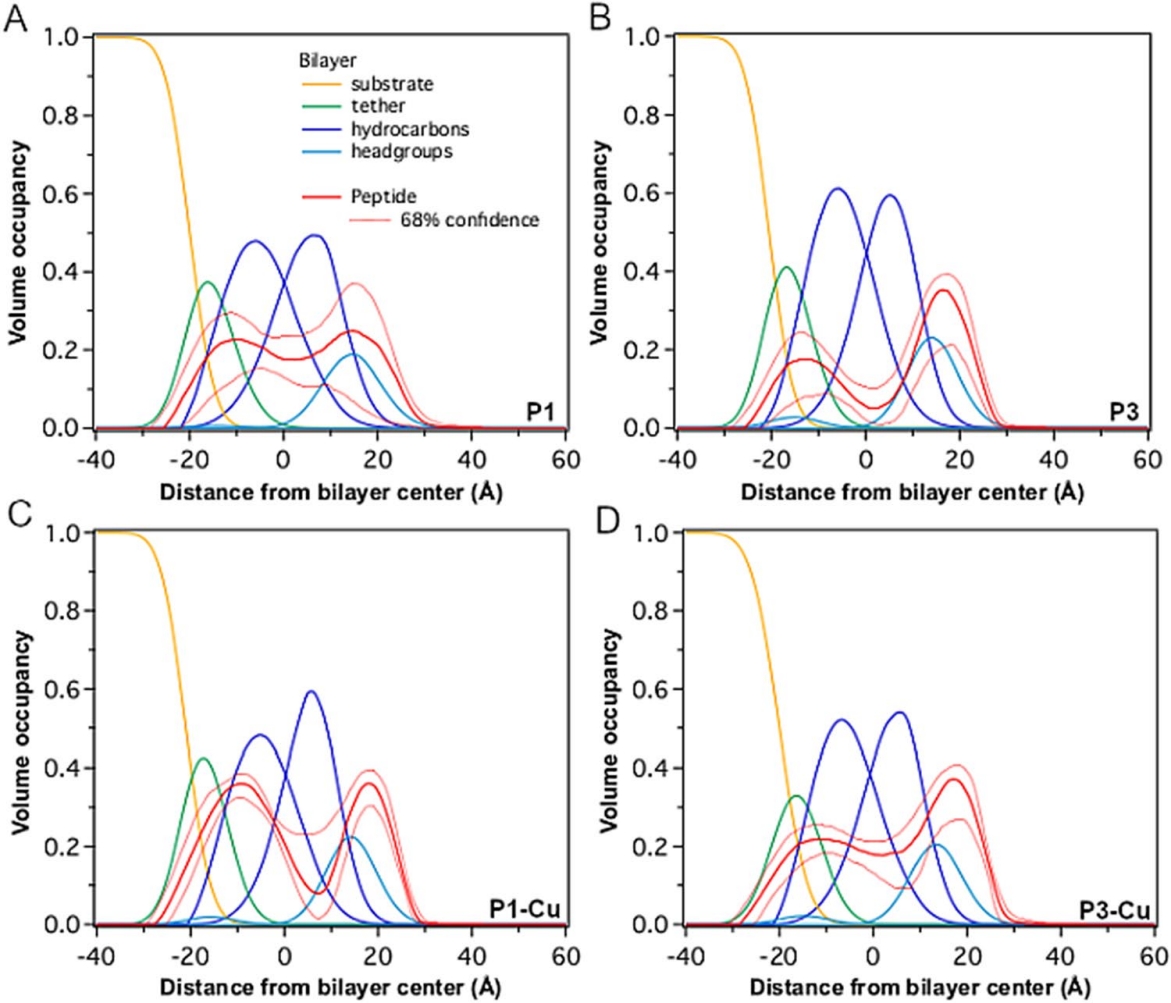
Neutron reflectometry of P1 and P3 in the apo- and holo-states. The time and ensemble average spatial profiles of the various components of the tBLM (Fig. 2A) are shown as projections on the normal to the bilayer surface for (**A**) P1, (**B**) P3, (**C**) P1-Cu^2+^, and (**D**) P3-Cu^2+^. The peptides were metallated using a 1:1 stoichiometric amount of CuCl_2_. The inner bilayer leaflet is attached to the gold coated substrate (yellow) via molecular tethers (green). The outer leaflet of the bilayer is exposed to the aqueous compartment from which each peptide solution is injected.

As many other helical HDPs, P1 and P3 adopt α-helical structures in the membrane-bound state^56,57^. The high resolution peptide structures derived from solid state NMR^58^, together with structural parameters of the lipid bilayer^82,83^ and scattering length densities (SLD) of the membrane components^84^, were integrated into a model to fit the depth-dependent distribution of each peptide in the bilayer. The best model fits were used to generate one-dimensional component volume occupancy (CVO) profiles^85^ (Fig. 4); these provide a way to quantify the fraction of the volume that is occupied by a particular molecular component as a function of the distance along the bilayer normal. For both P1 and P3 run at 3 µmol/L, peptide-to-lipid ratios (P/Ls) determined using the CVO profiles are close to 1/8 (see Table S1). This shows that the cationic P1/3 peptides freely bind to and robustly incorporate into the POPC bilayers, even in the absence of anionic lipids. Such a high P/L, as determined here, was previously associated with a regime of transition from surface-bound to tilted states and the subsequent formation of peptide-lined transmembrane defects^56^.

It is important to note that the CVO profiles represent a time and ensemble average of the bilayer-peptide assemblies (Methods). In light of this, the profiles show that after initial partitioning into the outer (distal) bilayer leaflet from the aqueous injection compartment above it (Fig. 2C), P1 and P3 distribute into the two tBLM leaflets to different extents. While apo-P1 spreads out evenly between the two lipid leaflets and across the hydrocarbon region, apo-P3 remains predominantly confined to the headgroup region of the outer leaflet, unable to transition as efficiently as P1 into the hydrocarbon region and the inner (substrate-proximal) leaflet (Fig. 4A, B).

Next, we observed the effect of metalation on the distribution of P1/3 in the tBLM. While P1-Cu^2+^ shows a slightly higher accumulation in the inner leaflet compared to its apo-form (Fig. 4A, C), P3-Cu^2+^ remains preponderantly confined in the outer leaflet (Fig. 4B, D). However, P3-Cu^2+^ shows a deeper insertion, by 2 Å, into the hydrocarbon region compared to apo-P3, based on the calculated center-of-mass (COM) positions. The COM positions for each peptide, with respect to the bilayer center, are: P1: 2 ± 2 Å; P1-Cu^2+^: 1 ± 1 Å; P3: 5 ± 2 Å; and P3-Cu^2+^: 3 ± 1 Å. We note that these COM positions correspond to the average over the ensemble of all peptide molecules in the bilayer. Since the ensembles are characterized by broad distributions extending over both leaflets, it is important to consider the profile distributions of the peptides in the bilayer (Fig. 4A–D) and not just the positions of their COMs. The calculated relative amounts of each peptide in the inner bilayer leaflet, including the sub-membrane aqueous space (Fig. 2C1), are: P1:(45 ± 7) %, P3:(37 ± 6) %, P1-Cu^2+^ (54 ± 6) %, and P3-Cu^2+^ (39 ± 5) % (Table S1). For these calculations, we note that we used boundaries for the lipid bilayer regions based on the convention established by White and Nagle^82,83^.

Overall, the NR results indicate that P1 has an inherently higher ability to populate the hydrocarbon region of the bilayer and to cross the membrane barrier in both the apo- and holo- states. The presence of Cu^2+^ has a more consequential impact on P3, boosting its propensity to insert in the hydrocarbon region. In combination with the SPR/EIS studies, these structural results reveal that there is a direct correlation between the increased potency of Cu^2+^-bound P1 and P3 against cancer cells (Table 1) and their ability to insert and overcome the bilayer barrier.

### Characterization of P1, P3, P1-Cu^2+^, and P3-Cu^2+^ in terms of bilayer conformations and disruptive effects to the bilayer structure

**Figure 5:**
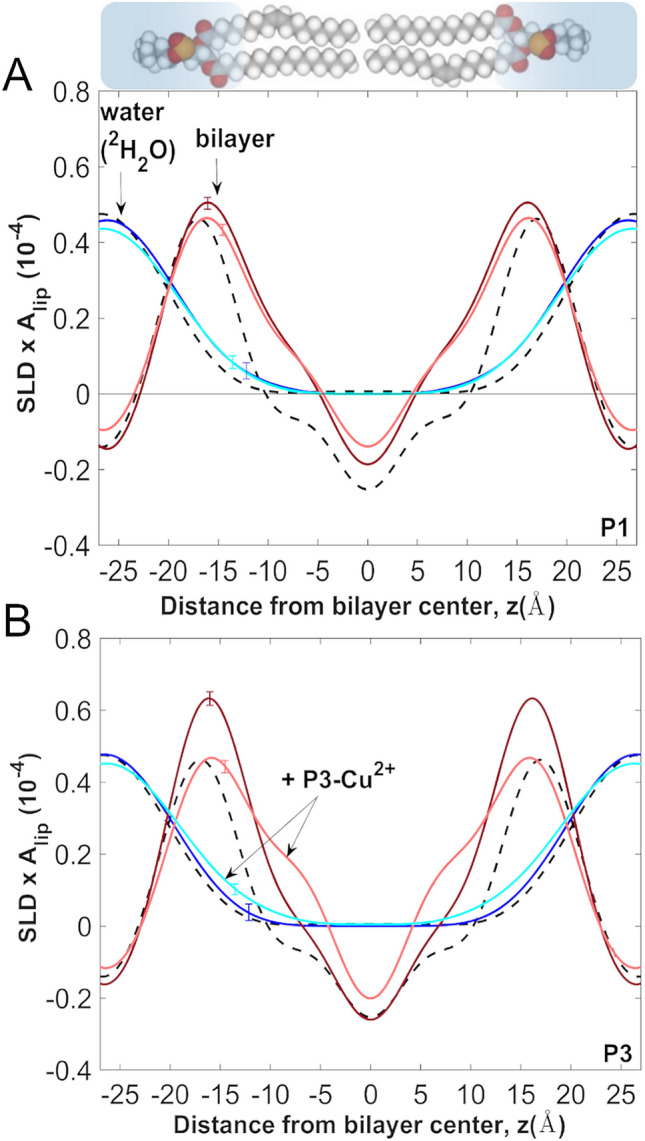
Bilayer scattering length density profiles from neutron diffraction on oriented lipid multilayers. (**A**) The scattering length density (SLD) profiles of the bilayer are shown as projections on the bilayer normal (z-axis) for POPC (black), P1/POPC (dark red), and P1-Cu^2+^/POPC (pink). The peptides were metallated using a 1:1 stoichiometric amount of CuCl_2_. The corresponding water profiles, determined from H_2_O/^2^H_2_O contrast are overlaid for POPC (black), P1/POPC (dark blue), and P1-Cu^2+^/POPC (light blue). Peptides distribute equally on both sides of the bilayer during their incubation with liposomes and deposition on the substrate (see Methods). (**B**) Same as in (**A**) but for P3. All oriented samples were prepared in POPC at P/L = 1:25, and measured at 23 °C and 93% relative humidity achieved by using the vapor phase of saturated salt solutions. The small error bars on the curves represent the uncertainty in the profiles, which were calculated using a 95% confidence interval in the Monte-Carlo sampling of the structure factors^87^. Structure factors and standard deviations are given in Table S2. All profiles were determined on a per-lipid scale using structure factors calibrated to reflect the composition of the unit cell and without explicitly determining the area per lipid^87^.

Following the NR experiments, we used ND to compare the effects of P1, P3, P1-Cu^2+^, and P3-Cu^2+^ on the bilayer structure and water distribution (Fig. 5). We worked at a P/L of 1:25 since we previously established that this value yields samples where the peptides are fully absorbed into the bilayer^56^. Because the multilayer samples are prepared by incubating liposomes with the peptides and the liposomes fuse once they are deposited on the glass substrate, the peptide molecules homogeneously populate both sides of the bilayer. The strong diffraction obtained from the multilayer systems allows us to analyze in greater detail the preferred position and conformation of each peptide in the bilayer^57^, together with perturbative effects of the peptides on the membrane structure.

Figure 5 shows the centro-symmetric bilayer SLD profiles of POPC bilayers (with and without peptides) projected on the normal to the bilayer (z-axis). The most prominent features are the phospholipid headgroups of the two opposing bilayer leaflets (high SLD) and the hydrocarbon region in the middle (low SLD). Incorporation of the holo-form of each peptide leads to significant density redistributions, seen here as changes to the bilayer profile (Fig. 5A, B) relative to the undisturbed POPC bilayer. These changes are indicative of pronounced structural perturbations when the peptides become integrated in the bilayer, including a measurable thinning of the bilayer, often associated with the action of membrane–active peptides. The thinning is seen here as a reduction in the repeat distance (*d*) in the multilayered samples (Fig. 2E) with P1/3, compared to the unperturbed POPC. The values for *d* and their uncertainties, calculated from the positions of the Bragg peaks (Methods), were found to be: POPC: 53.5 ± 0.04 Å; + P1: 52.2 ± 0.1 Å; + P1-Cu^2+^: 52.6 ± 0.1 Å; + P3: 52.9 ± 0.1 Å; and + P3-Cu^2+^: 52.7 ± 0.2 Å.

The contrast in the behaviors of P1 and P3 upon metallation is clearly discernable in the density profiles (Fig. 5). We observe that while metallating P1 has little consequence on the density profile of the peptide/bilayer ensemble (Fig. 5A), it has a quite dramatic effect on P3, since P3-Cu^2+^ produces a clear density shift compared to apo-P3 (Fig. 5B). As indicated by the raised SLD in the center of the bilayer and the broader profile of the lipid headgroup region, this density shift is due to the dual effect of P3-Cu^2+^ migrating closer to the bilayer center and the lipid headgroups being pulled in by the peptide through hydrogen bonding^56^. To show how the water distribution relates to these structural changes, we specifically determined the water profiles in the POPC bilayer (Fig. 5A, B, blue shades) by using H_2_O/^2^H_2_O exchange (deuterium contrast) and ND^86–88^ (Methods). As the charged peptide helices insert in the bilayer, not only do they deform the bilayer surface by engaging lipid headgroups, but they also pull in water molecules, seen here as an extension of the water profile into the hydrocarbon region (Fig. 5A, B). The water penetration is most pronounced for the Cu^2+^-bound P1/3, indicating that the metal ion increases the water penetration, an effect that correlates with the increased conductance seen in the EIS measurements presented above.

Similar bilayer density redistribution effects are observed when the peptides are metallated with Ni^2+^ (Fig. S6) instead of Cu^2+^ (Fig. 5). This shows that, broadly speaking, the two metal ions similarly modulate the interactions of the peptides with the bilayer. Importantly, in further control experiments, we found that the Cu^2+^ ions by themselves (without peptides) have a completely different effect on the bilayer structure (Fig. S5). In contrast to the peptide- Cu^2+^, the free metal ions cause an increase in the repeat spacing (*d*), which is most likely due to the binding of the hydrated ions to the lipid headgroups^89,90^, causing further water accumulation at the membrane surfaces and a broadening of the inter-bilayer space.

Based on the results mentioned above, the bilayer structural perturbations that we observe in the presence of holo-P1 and -P3 are exclusively due to the complexation of the metal ions with the peptides and the complexation affects the interactions of P1 and P3 with the lipid bilayer. In particular, the resulting net loss of one positive charge at the amino end of the peptide upon metallation very likely facilitates the bilayer insertion of the metallated peptides^63,64^. Given that metallation more dramatically impacts the IC_50_ and membrane conformation of P3 compared to P1, we further examined P3-Cu^2+^ within the bilayer and used available 3D structures to characterize its orientation.

### Position and orientation of P3-Cu^2+^ within the lipid bilayer

**Figure 6:**
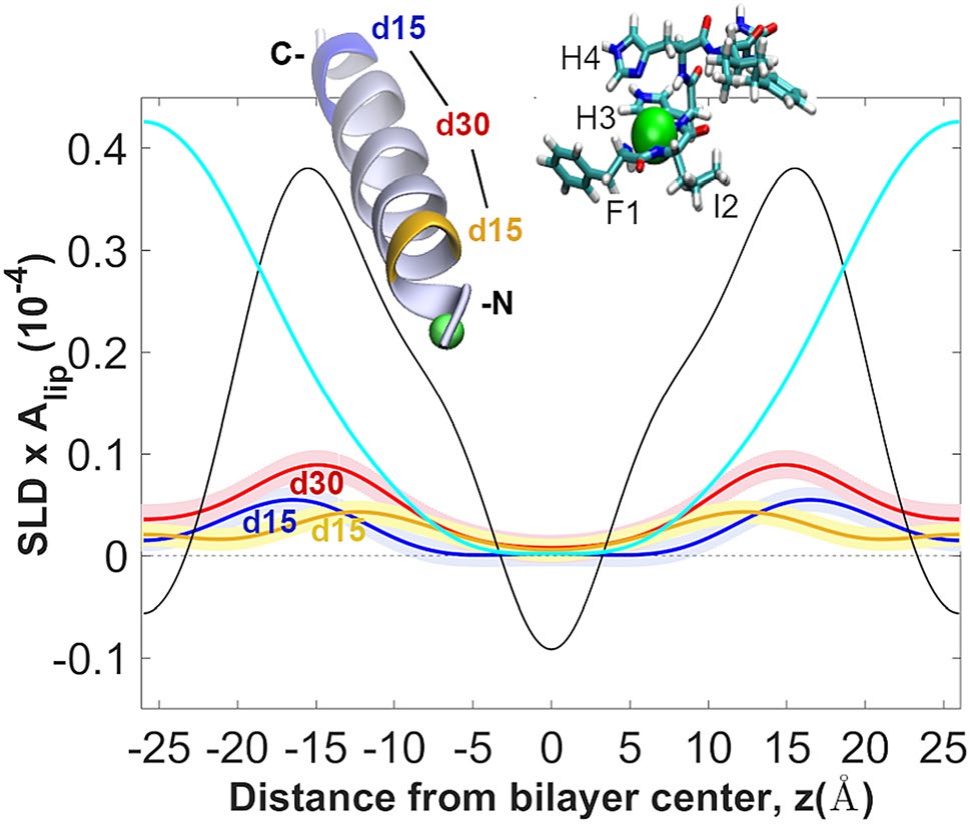
Positioning of P3-Cu^2+^ in the bilayer with ND and deuterium contrast. The SLD profiles of two groups of specifically deuterated amino acids near the N-terminus (I_5_^d10^F_6_^d5^; yellow) and C-terminus (F_19_^d5^L_20_^d10^; blue) of P3 were obtained, as well as their sum (red) (Methods). Each profile represents time and ensemble averages of each of deuterated groups in the thermally disordered bilayer. The overall profiles for the neat POPC bilayer with P3-Cu^2+^ (black) and the water distribution (blue) are overlaid on those for the deuterated groups. Measurements were done at P/L = 1:25, 23 °C, and 93% relative humidity. Uncertainty bands in the deuterium profiles (colored bands) were determined using a 68% confidence interval in the Monte-Carlo sampling of the measured structure factors^87^. The inset show representative average conformation of the P3 α-helix in the bilayer, using a 3D structure derived from the NMR structure (PDB ID # 6PEZ)^58^ and the predicted structure of the Cu^2+^-bound ATCUN motif based on density functional theory calculations^64^. The orientation of the α-helix in the bilayer is derived from the diffraction SLD profiles. The two deuterated sites are shown with yellow and blue; the Cu^2+^ ion (green sphere) is shown at a larger than true scale for better visibility.

In earlier investigations, we specifically deuterated P1 and P3 near their N-terminus (positions 5–6) and C-terminus (positions 19–20) and determined their deuterium profiles by ND^56^. Using the 3D structures of the membrane-bound peptides determined by solid-state NMR and the specific deuterium profiles we solved the orientations of each peptide in the bilayer in terms of tilt angle and insertion depths of the deuterated N- and C-terminal regions. Here, we employed a similar approach on P3-Cu^2+^ bound to POPC (Fig. 6).

As shown in Fig. 6, each of the two deuterated groups on the peptide define a broad, Gaussian-shaped distribution, when projected on the z-axis. The N-terminal group falls closer to the bilayer center than the C-terminal group. The distance between the maxima in the deuterium profiles of the two groups, which are both in the α-helical region of the peptide is 4.0 ± 0.4 Å. The distance between the COMs of these two groups along the α-helical axis, is 19.1 ± 1.2 Å. This was calculated by using the ensemble of structures previously solved by solid-state NMR for the apo-form (PDB ID # 6PEZ) and averaging their positions^58^. Based on these combined values, the estimated tilt of P3-Cu^2+^ is 78° relative to the normal to the bilayer (z-axis).

While we find the tilt of P3-Cu^2+^ in POPC to not be particularly pronounced, an important observation of this analysis is that the peptide is oriented so that the Cu^2+^-bound N-terminus is more inserted into the hydrocarbon region than the C-terminus (Fig. 6). This may appear surprising given the polar nature of the ion bound to the ATCUN motif. However, we also note that this peptide conformation is accompanied by a broader water distribution that extends into the hydrocarbon region (Figs. 5B and 6), trailing the Cu^2+^-bound ATCUN motif. Thus, we can infer the metal ion remains some degree of hydration in the bilayer interior, increasing water accessibility to the membrane core. The water penetration in the hydrocarbon is less pronounced for the undisturbed POPC bilayer, as well as for apo-P3/POPC system (Fig. 5B). We observe that P3-Cu^2+^ not only opens water accessibility into the hydrocarbon core, but the metal ion is located near the double bond of the oleic chain of POPC. Given the ability of Cu^2+^ to act as a catalyst for redox reactions, we decided to investigate whether the membrane-disruptive effects of the peptide go beyond the physical perturbations shown here and include the chemical oxidation of unsaturated fatty acids.

### Oxidation of linoleic acid double bonds in lipid vesicles in the presence of Cu^2+^, P1, P3, P1-Cu^2+^, and P3-Cu^2+^

**Figure 7:**
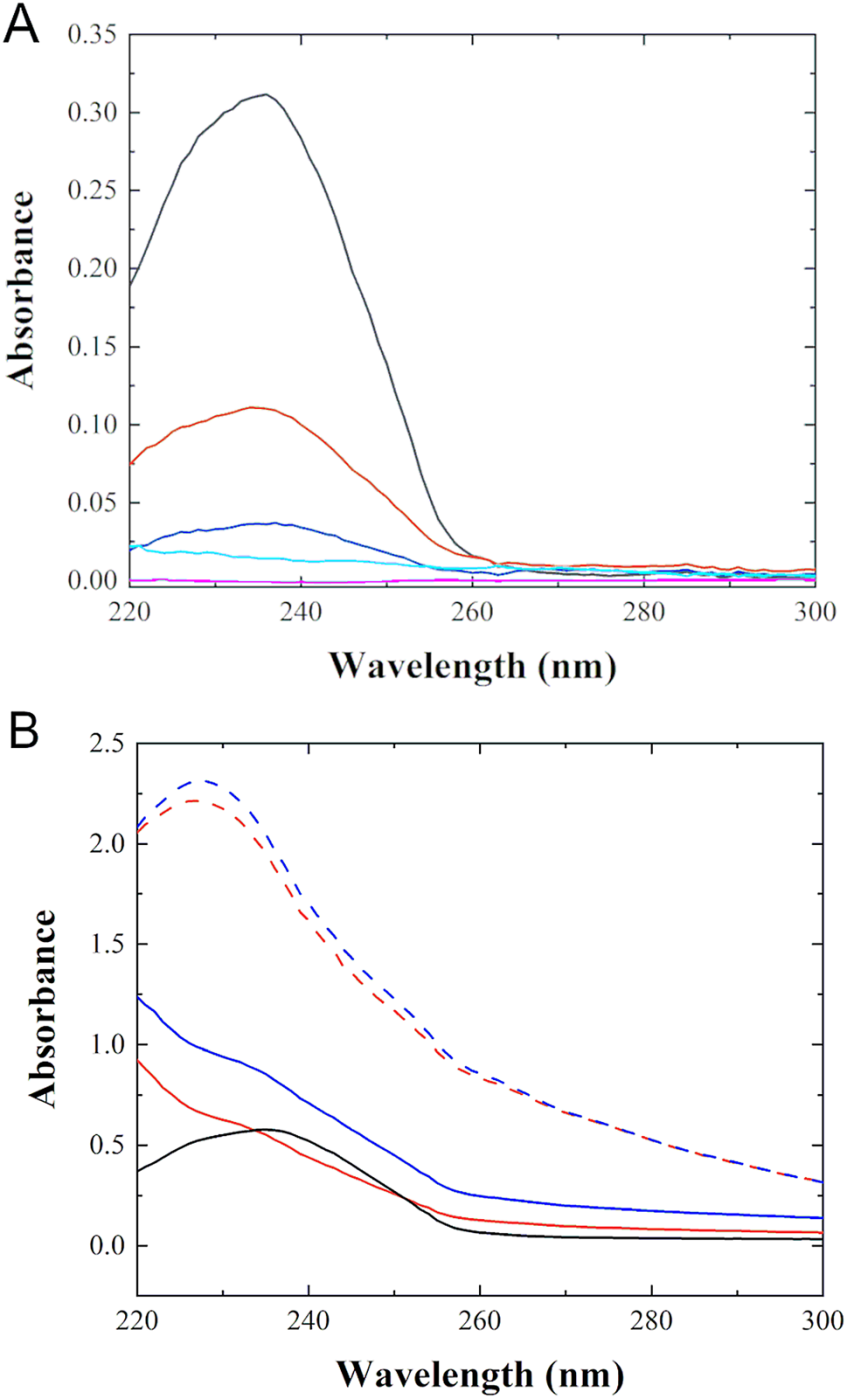
Absorbance measurements from vesicles containing a polyunsaturated lipid exposed to Cu^2+^ and piscidin bound to Cu^2+^. SUVs made of 3:1 DLPC/POPG were prepared and exposed to different forms and amounts of Cu^2+^ and measured by UV spectrophotometry after 24 h of exposure. (**A**) Samples containing SUVs at a fixed lipid concentration of 0.50 mg/mL (640 µmol/L) were mixed with various amounts of CuCl_2_, as follows: Cu^2+^/L = 1:2 molar ratio (black), Cu^2+^/L = 1:8 (red), Cu^2+^/L = 1:32 (blue), BHT/L = 1:100, and Cu^2+^/L = 1:8 (cyan) and lipid alone (magenta). (**B**) SUVs at a concentration of 0.20 mg/mL (260 µmol/L) were studied in the presence of the peptides at P/L = 1:10, corresponding to a peptide-Cu^2+^ complex concentration of 26 µmol/L. P1-Cu^2+^ (blue, dashed); P3- Cu^2+^ (red, dashed); P1 (blue); P3 (red); lipid alone (black). The source of free Cu^2+^ (CuCl_2_) was the same as that used for metallating P1 and P3. Measurements were taken in triplicates at 24 h after exposure. The spectra were corrected for the background signals from CuCl_2_ and the lipids at time zero as explained in the Methods. Uncertainties are smaller than the line thicknesses. Strong absorbance at 234 nm after 24 h of exposure points at the formation of lipid oxidation products, a result confirmed by mass spectrometry (Fig. S7).

It has long been known that certain divalent metal ions, especially iron and copper, promote lipid peroxidation (oxidative deterioration of polyunsaturated fatty acids), a mechanism that has been strongly associated with cellular injury and death^91^. Divalent transition-metal ions participate in Fenton-like chemical reactions produces ROS (e.g., hydroxyl radicals, superoxide) that can oxidize double bonds within the membrane^89^. Here, to learn about the possible piscidin/Cu^2+^-mediated oxidation of double bonds in lipid bilayers, we used absorbance and mass spectrometry to measure the formation of the oxidation products in small unilamellar vesicles (SUV) constituted of di-linoleoyl-*sn*-glycero-3-phosphocholine (DLPC) and POPG mixed in a 3:1 molar ratio and prepared at a concentration of 0.50 mg total lipid/mL. We first tested the ability of free Cu^2+^ to oxidize the double bonds within the liposomes. Increasing amounts of Cu^2+^ for a fixed amount of lipids (expressed as Cu/L, the molar ratio of Cu^2+^ to total lipid) were mixed with the SUVs and the absorbance was measured at λ = 234 nm (Fig. 7A) (corresponding to λ_max_ for the conjugated dienes that appear in lipid oxidation products)^92,93^. Because molecular oxygen (O_2_) found in the atmosphere readily dissolves in the bilayer, no exogenous oxidant is needed to initiate the oxidation of the double bonds^94^. As shown in Fig. 7A, a dose–response in oxidation yields is observed after 24 h with increasing Cu/L ratio. Similar results have been reported before on the oxidation of DLPC upon pre-incubation with Cu^2+^ for 24 h^95^. The Cu^2+^-catalyzed reactions were also measured in the presence of butylated hydroxytoluene (BHT), a free radical scavenger that interrupts the chain oxidation process^96^ (Fig. 7A). We see that BHT added in a proportion of 1 to 100 lipids stops lipid oxidation since the signal from the lipids/CuCl_2_/BHT sample is negligible at 234 nm.

It is important to note that divalent transition-metal ions can bind to both negatively-charged and zwitterionic lipid headgroups with high affinity, especially when amine moieties are present^89,90^. While it is difficult from the present studies to identify whether the free Cu^2+^ ions experience differential affinities for the PG and PC headgroups, our diffraction data (Fig. S5B) indicate that free Cu^2+^ binds to the headgroup region and causes lipid separation, presumably through a preferential segregation of the ions with the negatively-charged PG groups. This is consistent with our recent solid-state NMR data showing that Ni^2+^ preferentially interact with PG headgroups in PC/PG binary mixtures^63^. Due the sequestration of the Cu^2+^ on the membrane surface, it is conceivable that oxidizing agents that are generated near the membrane surface diffuse through the membrane, thus gaining access to the double bonds.

We compared the effect of free Cu^2+^ ions to that for P1 and P3, in both the apo- and holo- forms. As expected, apo-P1 and apo-P3 show little lipid oxidation (Fig. 7B), with the peak at 234 nm being swamped by a significant scattering contribution. This was consistent with our observation that the vesicle solutions show a faint turbidity upon peptide incorporation, even in the very dilute lipid solutions used to collect these data (0.20 mg/mL). However, samples containing the Cu^2+^-bound peptides display a prominent peak at 234 nm. Given that piscidins can serve as a Cu^2+^ carrier in the bilayer interior, according to our ND and NR results, it is concluded that the peptides facilitate the access of Cu^2+^ to the oxidation-prone double bonds, accelerating membrane damage. As revealed by mass spectrometry (Fig. S7), the lipid oxidation leads to fragmentation of the acyl chains and such a chemical damage could ultimately cause loss of bilayer structural integrity^97–99^.

## Discussion

The most compelling result from our SAR studies on the host defense metallopeptides P1 and P3 is the boost in both function and membrane insertion that is elicited by Cu^2+^-coordination. This is significant because it identifies metallation as a chemical modification that can improve the biological activity of this family of HDPs, several of which have already proven to be promising templates for the development of novel strategies to treat infections, inflammation, and cancer^7,8,15–22^. Specific valuable features of P1 and P3 include their broad spectrum activity in the micromolar and sub-micromolar (for P1) range, anti-inflammatory properties, and pH-resiliency in the physiological range^33,47,56,100–102^. P1, which has been more tested in vivo than P3, has very good pharmacodynamics and pharmacokinetics characteristics^61,62,102^. The versatility of these HDPs derive partly from the abundance of histidine residues that endows them with buffering capacity as well as the ability to bind and carry metal ions (Cu^2+^/Ni^2+^) to their cellular site of action^29,59,63,64^. Bound to each other, the Cu^2+^ ion and piscidin become co-localized in cellular compartments. In the natural setting of the fish, this corresponds to the phagosomes of immune cells and the extracellular environment upon degranulation^103^.

To the best of our knowledge, this is the first study to report the cytotoxicity of P1-Cu^2+^, P3, and P3-Cu^2+ ^on cancer cells. Apo-P1 was previously known to have anti-cancer activities^39^ but the effects of complexation with Cu^2+^ on this function had not been reported. We formerly showed that such metallation increases the direct antimicrobial potencies of P1 and P3 through effects that include the physical and chemical disruption of membranes and DNA^59,63^. This led us to assume that binding to Cu^2+^ could also enhance their cytotoxicity against cancer cells. The improved ability of piscidin to kill cancer cells upon Cu^2+^-coordination, as shown here, can be rationalized in part by examining peptide-bilayer interactions at the molecular level. In our earlier studies of apo-P1 and -P3, we demonstrated the abilities of the peptides to change their conformation from an unstructured form in solution to an amphipathic α-helix upon contacting the phosphate-containing groups of detergents and phospholipids^53–55,57,104^. This conformational change is induced by not only anionic but also zwitterionic membranes because the hydrophobic effect plays an important role in driving the partitioning of these peptides into amphipathic lipid membranes^105–107^. Using oriented CD, oriented-sample solid-state NMR^57,58,79^, ND^56^, and extensive all-atom MD simulations^56,57,108,109^, we found that P1 and P3 orient their amphipathic α-helices at an angle that depends on their concentration and the composition of the membrane. At low concentration, the peptides lie almost parallel to the bilayer plane. At higher concentrations, they become tilted and elicit membrane permeabilization. Lower P/L thresholds for tilting were observed for P1 than P3, and for zwitterionic than anionic bilayers, most likely because the anionic lipids retain the peptide on the bilayer surface^56,58,79^.

Here, we show that metallation with Cu^2+^ dramatically boosts the ability of P1 and P3 to kill cancer cells and produce conductive defects through otherwise electrically sealing model membranes. Indeed, our SPR/EIS measurements performed on a POPC-tBLM bilayer platform detected an immediate increase in conductance (drop in resistance) starting with apo-P1 at a concentration of 0.2 µmol/L. In the case of apo-P3, this response had a threshold of 0.5 µmol/L and was delayed by several minutes. Likewise, cancer cells were much more responsive to apo-P1 than apo-P3. Bound to Cu^2+^ before being added to cancer cells, P1 and P3 experienced IC_50_ values enhanced by a factor of up to ≈2 and ≈7 based on the data in Fig. 1, respectively, and this correlated well with increased bilayer conductances, as measured by SPR/EIS.

Our NR measurements done using the same supported bilayer as for the SPR/EIS experiments revealed the localization preferences of P1 and P3 in the bilayer milieu. Upon partitioning from the water phase into the bilayer outer leaflet (distal to the substrate), apo-P3 adopts a more superficial bilayer position than apo-P1. However, both peptides migrate to deeper locations in the hydrocarbon core when bound to Cu^2+^. Changes in the P1 distribution between the apo- and holo-forms have been difficult to assess because both states exhibit a high propensity for insertion in the hydrocarbon region. Nevertheless, while P1 remains broadly confined to the hydrocarbon core, metallation appears to induce a change in its bilayer position so that its COM moves closer to the inner leaflet. This suggests that complexation with the metal ion confers P1 with an increased ability to transfer from one side of the bilayer to the other.

In comparison to P1, P3 responds to Cu^2+^-complexation much more dramatically since its IC_50_ value improves by a factor of ≈7 versus ≈2 for P1 according to the data shown in Fig. 1. As shown by the NR and ND profiles, this effect correlates with Cu^2+^-binding having a more pronounced effect on P3 than P1 in terms of how the peptides get redistributed toward the bilayer center. ND offers additional information when used in conjunction with site-specific deuteration of P3: the Cu^2+^-carrying N-terminus of P3 is found to be strikingly more inserted in the hydrocarbon region than its C-terminus. For comparison, a similar orientation was reported for apo-P3 in POPC/POPG bilayers, albeit at a more pronounced tilt angle^56^, suggesting that both the presence of the metal ion and lipid composition can subtly influence the bilayer conformation of the peptide in terms of tilt and depth of insertion. The deeper insertion achieved by piscidin upon metallation can be explained by the loss of one positive charge at the amino end of the peptide when the metal ion binds^59,63,64^. Our earlier combined NMR and DFT structural investigations of P3-Ni^2+^ bound to 3:1 PC/PG suggested that the bulky and hydrophobic I2 and H4 side chains surround the metal ion to facilitate its insertion into the hydrocarbon region^64^. As revealed by our ND data and our lipid oxidation assays in model membranes, the inserted ATCUN-Cu^2+^ not only opens the bilayer for water penetration, but also appears to promote oxidation in the surrounding lipid. This dual ability to physically and chemically alter bilayers adds to the multifaceted actions that piscidins may be able to exert within living cells. As we previously showed, metallation of both P1 and P3 results in enhanced MICs and lipid peroxidation effects in bacterial cells^59^. In model membranes, the permeabilization effects of P1 are improved by metallating the peptide as well as adding oxidized lipids to the membrane^63^.

With regard to cancer cells, Lin et al. showed that apo-P1 exerts apoptotic effects on HT1080 cells at low concentrations while its mechanism of action is necrotic at higher doses^39^. Cheng et al. more recently observed that apo-P1 also has apoptotic effects on osteosarcoma (bone) cancer cells^78^. More specifically, the peptide enhanced the formation of mitochondrial ROS, resulting in oxidative damage. Apoptosis as a mechanism of cell death has been observed for other ACPs, including the synthetic peptide A_9_K and a derivative of magainin-2^19^. This apoptotic cell death pathway is consistent with our microscopy studies presented here. Indeed, we found that P1 and P3 are cell penetrating at low concentrations and reach the mitochondria. Once in this location, it is plausible that they disrupt the mitochondrial membrane and initiate apoptosis. From these results, we would infer that at low concentrations, P1 and P3 are more disruptive to mitochondrial than plasma membranes. This is expected because of the differing lipid compositions of these organelles, with the former containing the anionic lipid cardiolipin (CL) that is well known to attract cationic HDPs^7,15,17,110^. Interestingly, we previously showed that P1 and P3 leverage membrane heterogeneity to disrupt membranes containing phosphatidylethanolamine (PE), another lipid abundant in mitochondrial membranes^58^. Further supporting the case that P1 and P3 interact favorably with mitochondrial membranes is the research by Tin et al. on tilapia P4, TP4^43^. The authors showed that this piscidin, which was successfully used in vivo against triple-negative breast cancer, exhibits strong affinity for the mitochondrial membrane of cancer cells. Interestingly, they found evidence for necrotic cell death, with damaged mitochondria leaking calcium and experiencing disrupted calcium homeostasis and signaling.

Several peptides, including pleurocidin that is related to piscidin, use ROS formation to induce cell death via apoptosis or inhibition of nucleic acid synthesis^111^. Another cationic HDP with anti-cancer property, lactoferricin B interacts with the mitochondrial membrane, leading to the formation of ROS and inducing apoptosis^112,113^. Tilapia hepcidins also feature strong anti-cancer activity and the reduction of multi-drug resistance via a mechanism that involves ROS formation and the activation of a mitochondrial apoptosis pathway^114–117^.

Our study focused on showing that metallation enhances the cytotoxic effects of piscidins 1 and 3 on cancer cells and explaining this effect through a detailed characterization of peptide-lipid interactions. As a next step, it will be important to determine whether the mechanism of cell death by P1 and P3 bound to Cu^2+^ features apoptosis, as previously shown for apo-P1^39,78^. For instance, it would be insightful to use cell-based assays to determine whether the Cu^2+^-bound peptides release Cu^2+^ intracellularly (e.g., acidic endosomes) resulting in enhanced ROS formation and increased apoptotic effects compared to apo-P1 used at low concentration. Combined with biophysical studies using model membranes that incorporate lipids present in the outer leaflets of cancer cell plasma (e.g., PS, PE, cholesterol) and mitochondrial membranes (e.g., CL, PE), this could help shed light on the propensity of the peptides to bind to and disrupt one type of membranes over the other. In the case of P3-Cu^2+^, which is known to be more damaging to bacterial DNA than membranes^59,79^, there is also the possibility that part of its mechanism involves using Cu^2+^ to form radicals that damage mitochondrial DNA.

Mitochondrial targeting has attracted a lot of attention in cancer therapy^6,17,40,41,118–120^. One of the major reasons is that cancer cells are more sensitized than healthy cells to mitochondrial perturbations due to their metabolic reprogramming. Cationic molecules that can accumulate preferentially in mitochondria based on charge and membrane potential have been particularly sought after since they can help deliver cargoes with anticancer properties and induce cell death^17,18,40,118–120^. The potential to use cationic host defense metallopeptides to cross cell membranes and reach mitochondria to deliver a cargo, such as a redox center is worth further investigating based on our results with piscidin. In particular, the spontaneous ability of metallated piscidin to release Cu^2+^ within acidic compartments (e.g., late endosomes) could contribute to their considerable efficacies. From this perspective, peptides with different potencies could be designed by varying their cell penetrating properties and pH-dependent affinities for Cu^2+^.

We note that before anticancer piscidin peptides bound to Cu^2+^ can be safely used in vivo, their stability in blood plasma has to be assessed since toxic effects could occur if Cu^2+^ were released and/or transferred to plasma proteins before reaching cancer cells^121^. Miyamoto et al. recently made constructs of the ATCUN motif and octreotide, a tumor homing sequence^122^. In terms of blood plasma stability, the authors found that bulky residues within the ATCUN motif stabilized the Cu^2+^-peptide complexes. YYH-octreotide, the most stable construct in blood plasma was successfully used in vivo to image tumors by ^64^Cu positron emission tomography. In the case of piscidin, we recently showed using NMR and quantum calculations that favorable cation-π interactions are possible between the metal ion and the bulky aromatic residues in the N-terminal region of the peptide, resulting in a stabilizing effect for the metal ion^65^. Given that piscidin and YYH-octreotide have metal-binding regions similarly rich in aromatic residues, we anticipate comparable stability of their Cu^2+^-complexes in vivo. Additional studies with piscidin peptides are needed to verify this prediction.

## Conclusion

This work reveals that metallating HDPs is an effective strategy to enhance their anticancer properties through the expansion of their mechanism of action, so that both physical and chemical damage of membranes is achieved. In particular, our results from cell-based assays, electrochemistry and structural measurements clearly establish that coordinating Cu^2+^ to P3 tremendously enhances its cytotoxic effects on cancer cells and this effect correlates with a stronger ability of the holo- than apo-form to interact with model membranes. Since both P1 and P3 have cell penetrating properties at sub-lethal concentrations, they can not only use Cu^2+^-binding to enhance their bilayer penetrating abilities but also carry and release Cu^2+^ intracellularly, resulting in other disruptive effects such as ROS formation, lipid peroxidation, and the destabilization of mitochondrial membranes and/or DNA. As membrane-interacting Cu^2+^-binding peptides that have favorable biocompatibility properties and can reach mitochondria, piscidins could represent new opportunities in the development of novel mitochondria-targeting therapeutics to treat life-threatening diseases such as cancer.

## Methods

### Materials

Carboxyamidated P1 and P3, including the ^2^H-labeled forms, were chemically synthesized at the University of Texas Southwestern Medical Center. Once received, they were purified and lyophilized, as previously reported^57^. The peptides were dissolved in dilute HCl to substitute chloride for trifluoroacetate ions and dialyzed to remove excess salt, leading to 98% pure peptides^57,100^. Following reconstitution of the peptides in nanopure water, their molar concentrations were determined by amino acid analysis performed at the Protein Chemistry Center at Texas A&M. Metallation was done in a 1:1 molar ratio between the peptide and metal using aqueous CuCl_2_ (Hampton Research, Aliso Viejo, CA). The pH was carefully adjusted to 7.4 since protons are released by the peptide upon metal binding^64^. MTT reagent (Thiazolyl Blue Tetrazolium Bromide) was purchased from Sigma-Aldrich. Lipids, 1-palmitoyl-2-oleoyl-*sn*-glycero-3-phosphocholine (POPC), di-linoleoyl-*sn*-glycero-3-phosphocholine (DLPC) and 1-palmitoyl-2-oleoyl-*sn*-glycero-3-phosphatidyl-*rac*-glycerol (POPG), were obtained from Avanti Polar Lipids (Alabaster, AL).

### Cell cultures

A549 human lung cancer cells were obtained from ATCC (ATCC CCL-185) and were maintained in Dulbecco’s Modified Eagle’s Medium (DMEM-F12, Lonza, catalog no. 12-719F) supplemented with 10% heat inactivated FBS and 1% Penn-Step. The HT1080 and MRC-5 cells were purchased from ATCC (catalog no. CCL-121 and CCL-171, respectively) and grown in Eagle’s Minimum Essential Medium (ATCC, catalog no. 30-2003TM) that was supplemented with FBS (ATCC, catalog no. 30-2021TM) such that the medium contained 10% FBS. The MDA-MB-231 and HeLa cells were obtained from ATCC (ATCC, catalog no. HTB-26 and CCL-2, respectively) and maintained in DMEM supplemented with 10% FBS. All cell cultures were grown at 37.5 °C in a humidified 5% CO_2_ atmosphere.

### MTT/MTS assays on cancer cells to evaluate the cytotoxicity of P1 and P3 in the apo- and holo-states

The cytotoxicity assays followed well established procedure, with the details given in the Supplementary Information. The assays were performed in triplicates using 96 well plates Cell viability was calculated using the equation: % viability = [(AT-AB)/(AC-AB)] × 100, where AT is the absorbance of the treatment well, AB is the average background absorbance measured from three wells containing only media, and AC is the absorbance of the positive control well, which received only 100 µL of media and 40 µL of MTT solution (i.e. no peptide). Averages and error bars were calculated as the mean + / − standard deviation based on triplicates. The IC_50_ values were obtained by fitting the percentage viability (% viability) vs Log (M) concentration of peptides to generate inhibitor dose response curve using the GraphPad Prism software. The curves were fitted using a least-squares minimization method. Individual data points on the graphs are provided as the mean (± standard deviation) from the triplicate % viability values. The GraphPad Prism algorithm converted the logIC_50_ into IC_50_ and used the standard error of the mean on the logIC_50_ to yield the 95% confidence interval for the logIC_50_ and IC_50_.

### Laser confocal microscopy

HeLa and MD-MBA-231 cells were propagated in DMEM supplemented with 10% FBS and containing 200 U/mL each of penicillin and streptomycin. Incubation was done at 37 °C with 5% CO_2_. Then 20,000 cells were seeded into each well of a chambered cover glass and allowed to adhere overnight. The cells were exposed to 100 nmol/L MitoTracker, 5 µg/mL Hoescht 33,258 (for DNA staining), and 4 µmol/L FITC-labeled peptides for 20 min. The medium was then aspirated, the cells were washed with PBS before adding fresh medium. The cells were imaged on a Nikon A1R spectral confocal microscope. Images are shown as acquired without any post-imaging processing.

### Surface plasmon resonance (SPR)/electrochemical impedance spectroscopy (EIS)

SPR/EIS measurements were carried out on tethered bilayer membrane (tBLM) using a custom-built instrument previously described (Fig. 2B)^123,124^. Polished sapphire substrates were coated with a 5 Å layer of chromium followed by a 440 Å gold film by magnetron sputtering (Denton Vacuum LLC, Moorestown, NJ) at the nanoFab facility of the National Institute of Standards and Technology (NIST). Several POPC tBLMs were assembled on the gold surfaces using thiolated polyethyleneglycol linker molecules (1,2-di-O-myristyl-3-[ω-mercaptohexa(ethylene oxide) glycerol] (WC14) and β-mercaptoethanol (βME) at a ratio of 70:30 βME:WC14. The tBLMs were equilibrated in Tris buffer (20 mmol/L, 50 mmol/L NaCl, pH 7.4) and allowed to rest until both SPR and EIS signals were stable, before peptide injection. Two types of measurements were done. In the first type (Fig. 3), the peptide concentration was increased gradually, in steps of 0.1 μmol/L, and the SPR/EIS was monitored for 10 min after each addition. The peptide addition was repeated until the bilayer resistance dropped significantly. In the second type (Fig. S3), each peptide was introduced onto the tBLM-containing SPR/EIS cell at a fixed concentration of 3 μmol/L. After 10 min of incubation, the cell was rinsed with the buffer, and the SPR/EIS measurements were continued for another 10 min. In each case, intensity distribution versus pixel position on the camera chip was measured by SPR. The pixel positions of the SPR signal minima were plotted as a function of time. EIS data were recorded from 1 to 10 kHz at a rate of 1 spectrum per minute, resulting in a total of 22 spectra for each experiment. Each spectrum contained 100 data points. The Z-plot and Z-view software (Scribner Associates, Inc., NC) was employed for the impedance spectral collection and analysis. The simplest electrical circuit model (Fig. 2D) was used to fit the impedance data using a complex nonlinear least-squares minimization procedure based on a Levenberg − Marquardt algorithm implemented in the Zview software. The standard deviation in the fit parameters (e.g., capacitance and resistance) were calculated from the residuals of the fit (mean square error).

### Neutron reflectometry (NR)

NR measurements were conducted on POPC tBLMs supported on 3 inch diameter, 5 mm thick n-type Si:P[100] wafers (El-Cat Inc., Ridgefield Park, NJ). The wafers were cleaned and coated with Cr (≈20 Å) and Au (≈150 Å) at the NIST nanoFab facility. Coated substrates were immersed in a 7:3 (mol/mol) ethanol solution of HC18^125^ and β-mercaptoethanol at a total concentration of 0.2 mmol/L to form a self-assembled monolayer (SAM). Substrates were assembled in fluids cells^126^ and 5 mg/mL solutions of POPC vesicles were added to the dry SAM for 2 h. Afterwards, the system was flushed with pure water to complete tBLM formation. Before the addition of peptide, NR measurements of the tBLM by itself was carried out to verify the formation of a complete bilayer. Peptide in buffer (20 mmol/L Tris, pH 7.5), at a solution concentration of 3 µmol/L, was then injected in the aqueous compartment adjacent to the tBLM, allowed to incubate with the bilayer for 30 min, and then rinsed with buffer to remove any loosely bound peptide. For each sample, data were sequentially collected with D_2_O and H_2_O-based buffers. Buffer exchange was achieved by flushing ≈10 mL of buffer through the cell (volume, 1.3 mL) using a syringe. NR measurements were performed at the CGD-Magik reflectometer at the NIST Center for Neutron Research (NCNR)^127^. Reflectivity curves were recorded at room temperature, for momentum transfer values 0.01 ≤ *q*_z_ ≤ 0.25 Å^−1^. For data analysis, 1D-structural profiles of the substrate and the lipid bilayer along the lipid bilayer normal were parameterized using a continuous distribution model as described elsewhere^85^. Within such a model, spatial distributions of molecular groups along the bilayer normal are described with continuous component volume occupancy (CVO) profiles. Optimization of model parameters was performed using the ga_refl and Refl1D software packages developed at the NCNR^126^. All reflectivity curves of one data set were fitted simultaneously to the same model, sharing common fit parameters, for example for the solid substrate. A Monte-Carlo Markov chain-based global optimizer was used to determine fit parameter confidence limits^126^.

### Neutron diffraction (ND)

Oriented lipid multilayers samples of POPC and piscidin at P/L = 1:25 (Fig. 2E) were prepared as previously described^58^. The Cu^2+^-bound peptides were solubilized initially in pure water and equilibrated in sodium phosphate buffer (4 mmol/L phosphate buffer, pH 7.8). Then, they were mixed with POPC liposomes in the same buffer and allowed to incubate for 1 h before fusing on glass substrates. ND measurements on oriented lipid multilayers (Fig. 2E), probing the direction orthogonal to the bilayer plane, were acquired with the instrument CGD-MAGIk at the NCNR. The data were processed and analyzed as described before^86,128^. In short, bilayer structure factors were obtained as the square root of the integrated Bragg peaks, corrected for background, absorption and extinction, and their phases determined by deuterium contrast, using H_2_O/^2^H_2_O exchange^86–88^. Tables with structure factors can be found in Supplementary Information (Tables S2 and S3). The bilayer one-dimensional SLD profile was calculated by Fourier synthesis of the structure factors. The contrasts between deuterium-containing and natural abundance samples arising from the higher neutron scattering length of deuterium (b^2^H = 6.67.10^–5^ Å) with respect to hydrogen (bH =  − 3.74·10^−5^ Å) were used to parse out the specific profiles of the deuterated regions. The amplitudes of the profiles were determined to the best approximation using ^2^H_2_O for calibration^87^ and considering that there are 9.4 waters per lipid headgroup at 93% relative humidity and that the peptides bring in an additional 1.2 waters/lipid on average when the P/L is 1:25^56^. Repeat distances, *d*, (Fig. 2E) and their uncertainties were determined by a linear fit of the Bragg peak position versus diffraction order.

### Lipid oxidation measurements

Fresh vials of DLPC and POPG in chloroform were open and mixtures of the two lipids were prepared in an oxygen-free chamber. After the evaporation of chloroform under a stream of nitrogen gas, SUVs were produced by suspending dry lipids in pure deionized water at a concentration of 0.50 mg/mL, followed by sonication to obtain a highly clear solution. Oxidation of polyunsaturated lipid chains of DLPC in the presence of Cu^2+^, P1-Cu^2+^, or P3-Cu^2+^ were monitored based on the appearance of an absorbance peak at 234 nm. This peak was previously reported to represent the formation of lipid oxidation products (e.g. conjugated dienes)^92^. For samples containing SUVs and CuCl_2_, oxidation was initiated by adding CuCl_2_ to the freshly prepared SUVs (0.50 mg/mL), at final copper/lipid (Cu/L) molar ratios of 1/2, 1/8, and 1/32. Control samples (without copper) or in the presence of the antioxidant butylated hydroxytoluene (BHT) were also measured. Samples containing P1 or P3 in the apo or holo states were prepared in water at a final P/L = 1/10 and stabilized in a sodium phosphate buffer (4 mmol/L, pH 7.8) to preserve the complexation of the two peptides with Cu^2+^. The lipid concentration in the peptide-containing samples was 0.20 mg/mL. The buffer concentration was kept minimal to avoid turbidity effects of the lipid dispersions. Since it takes several hours to achieve significant oxidation, the degree of lipid oxidation was assessed for all samples after exposure to air for 24 h, by measuring absorbance spectra in the range of 200 to 300 nm with a Molecular Devices Spectra Max M5 plate reader. The absorbance values reported and their standard deviations were obtained by averaging values from three simultaneous, independent measurements. The spectrophotometer was blanked using CuCl_2_ diluted at concentrations equivalent to those used in the samples measured in Fig. 7. To correct for the background from unoxidized lipids, we subtracted from the averaged spectra the signal collected for the SUVs alone at time zero.

### Mass spectrometry

Aliquots of fresh DLPC were taken after opening the lipid vials, coordinating with the timeline of the oxidation measurements above. POPG was not used in order to avoid complicating the spectrum, unnecessarily. The lipid in chloroform was dried under a stream of nitrogen gas. Solubilized in water, CuCl_2_ was added to a Cu/L ratio of 1:2 and allowed to incubate for 20 h. Just before mass spectrometry measurements, the DLPC/Cu^2+^ samples were diluted in a 1:1 water/methanol solution to a final concentration of 100 µmol/L. Electrospray Mass Spectrometry (ESI–MS) was performed on an Orbitrap Elite (Thermo Fisher, San Jose, CA) in positive mode. Samples were directly infused into the mass spectrometer at a flow rate of 10 µL/min.

## Supplementary Information

Supplementary Information 1.

## Abbreviations

ACPs: Anticancer peptides
AMPs: Antimicrobial peptides
ATCUN: Amino terminal copper and nickel
BHT: Butylated hydroxytoluene
CL: Cardiolipin
CD: Circular dichroism
COM: Center of mass
CVO: Component volume occupancy
DLPC: Di-linoleoyl-*sn*-glycero-3-phosphocholine
EIS: Electrical impedance spectroscopy
FITC: Fluorescein isothiocyanate
HDPs: Host defense peptides
MDR: Multi-drug resistance
MIC: Minimum inhibitory concentration
MD: Molecular dynamics
ND: Neutron diffraction
NR: Neutron reflectometry
P1: Piscidin 1
P3: Piscidin 3
PDB: Protein data bank
PE: Phosphatidylethanolamine
P/L: Peptide-to-lipid ratio
POPC: 1-Palmitoyl-2-oleoyl-*sn*-glycero-3-phosphocholine
POPG: 1-Palmitoyl-2-oleoyl-*sn*-glycero-3-phosphatidyl-*rac*-glycerol
ROS: Reactive oxygen species
SAM: Self-assembled monolayer
SLD: Scattering length density
SARs: Structure-action relationships
SPR: Surface plasmon resonance
tBLM: Tethered bilayer membranes
TP4: Tilapia piscidin 4
