# Supplementary material for: Copper-binding anticancer peptides from the piscidin family: an expanded mechanism that encompasses physical and chemical bilayer disruption
Source: Sci Rep. 2021 Jun 16;11:12620. doi: 10.1038/s41598-021-91670-w (PMC8208971; doi:10.1038/s41598-021-91670-w)
Supplement: Supplementary file 1 — Supplementary Information 1. [file 41598_2021_91670_MOESM1_ESM.pdf]

Supplementary Information

Copper-binding Anticancer Peptides from the Piscidin Family: An Expanded Mechanism that Encompasses Physical and Chemical Bilayer Disruption

Fatih Comert[1], Frank Heinrich[2,3], Ananda Chowdhury[1], Mason Schoeneck[4], Caitlin Darling[5], Kyle W. Anderson[1,6], M. Daben J. Libardo[7], Alfredo M. Angeles-Boza[7], Vitalii Silin[1], Myriam L. Cotten[8*], and Mihaela Mihailescu[1*]

[1]Institute for Bioscience and Biotechnology Research, Rockville, MD 20850, USA. [2]Department of Physics, Carnegie Mellon University, Pittsburgh, PA 15213, USA. [3]Center for Neutron Research, National Institute of Standards and Technology, Gaithersburg, MD 20899, USA. [4]University of Rochester School of Medicine and Dentistry, Rochester, NY 14620, USA. [5]Department of Biological Sciences, Clemson University, Clemson, SC 29634, USA. [6]Biomolecular Measurement Division, National Institute of Standards and Technology, Gaithersburg, MD 20899, USA. [7]Department of Chemistry and Institute of Materials Science, University of Connecticut, Storrs, CT 06269, USA. [8]Department of Applied Science, William and Mary, Williamsburg, VA 23185, USA.

Correspondence: Prof. Myriam L. Cotten: mcotten@wm.edu; Dr. Mihaela Mihailescu: emihailescu@ibbr.umd.edu

Running Title: Piscidin-Cu^{2+}: Enhanced cytotoxicity and bilayer disruption

Supplementary information for MTT/MTS assays on cancer cells.

A549 cells were seeded in 96 well plates pre-coated with 0.1% porcine gelatin solution. Cells were seeded at the density of 20,000 cells/well in 200 µL of complete media and allowed to adhere for 24 h. P1 and P3 in the apo- and holo-forms were diluted in media from a single stock to generate 100 µL of solution at the final concentrations needed in the assay (1.21-38.88 µmol/L for P1; 1.25-40-12 µmol/L for P3). Each set of conditions was pipetted in triplicate in two plates, with one plate tested 24 h and the other at 48 h. For the characterization of cell survival, 40 µL of 5 µg/µL MTT reagent dissolved in 1X Dulbecco's Phosphate Buffered Saline (DPBS, ATCC, catalog no. 30-2200) was added per well and incubated for 3 h at 37ºC and 5% CO_2. Plain blanks (to account for spontaneous MTT precipitation) were obtained by plating 40 µL of the MTT reagent with an equal volume of media. Following the 3 h incubation, the media was carefully aspirated from the wells and the insoluble formazan product was solubilized by adding 100 µL of dimethyl sulfoxide and agitating the plate in a shaker for 10 min. To monitor the amount of live cells that could react with the MTT reagent, the solution was read at 560 nm (purple) in a Tecan Infinite M 1000 pro-plate reader. A similar approach was used to perform the MTS assay on the HT1080 cells (performed at Hamilton College) as well as MDA-MB-231 and HeLa cells (performed at the University of Connecticut). First, the peptide stocks were serially diluted in DPBS to 10X of their desired concentration in the wells (2- 12 µmol/L for P1; 2-30 µmol/L for P3). During peptide treatment of the cells (5,000 for HT1080), each well contained 90 µL of media and 10 µL of the peptide solution in DPBS. Second, cell viability was assessed after 24 h of treatment by adding 20 µL of CellTiter 96 AQueous One Solution Cell Proliferation Assay (MTS; Promega, Madison, WI, USA) and reading the absorbance at 490 nm after 2 h of incubation at 37.5 °C in a humidified 5% CO_2 atmosphere.

Table S1. Neutron reflectometry (NR): Selected fit parameter values from analyzing NR data sets collected after peptide injection. The amount of membrane-associated peptide is given as a volume surface density in units of Å^3/Å^2. The uncertainties associated with the fits are reported as one standard deviation (68% confidence level). Fit quality was estimated based on the reduced $\chi_v{}^2$ values for the fits. These values for the fits of the P1, P1-Cu^{2+}, P3 and P3-Cu^{2+} data sets were $\chi_v{}^2 = 1.2$, 1.1, 1.3 and 1.2, respectively.

Parameter	P1	P1-Cu	P3	P3-Cu
Tether thickness (Å)	7.6 ± 0.2	8.7 ± 0.1	7.7 ± 0.2	7.5 ± 0.1
Hydrocarbon thickness inner lipid leaflet (Å)	12 ± 1	13.5 ± 0.7	13 ± 1	11.7 ± 0.8
Hydrocarbon thickness outer lipid leaflet (Å)	11 ± 1	10.2 ± 0.7	10 ± 1	10.3 ± 0.9
Area per lipid, outer leaflet (Å^2)	86 ± 9	91 ± 7	90 ± 9	90 ± 8
Bilayer completeness (%)	99 ± 2	99 ± 1	98 ± 2	99 ± 1
Membrane-associated peptide (Å^3/Å^2)	10 ± 3	12 ± 2	8 ± 2	11 ± 2
Peptide-to-lipid ratio, P/L	1/(8±3)	1/(7±1)	1/(10±3)	1/(7±2)
Peptide fraction in headgroups total (%)	33 ± 9	34 ± 2	45 ± 6	37 ± 3
Peptide fraction in hydrocarbons total (%)	50 ± 10	46 ± 3	31 ± 8	37 ± 5
Peptide fraction in inner leaflet[a] (%)	40 ± 7	49 ± 6	32 ± 6	32 ± 5
Peptide fraction in outer leaflet[a] (%)	43 ± 6	32 ± 4	44 ± 6	43 ± 3

[a]Inner leaflet refers to the bilayer leaflet proximal to the gold substrate, while the outer leaflet is exposed to the bulk fluid compartment above the bilayer (injection compartment).

Table S2. Neutron Diffraction (ND) structure factors. Structure factors and standard deviations (SD) for POPC aligned multilayers in the presence of unlabeled peptides.

P1

F_H_2O	(SD)	ΔF_^2H_2O[A]	(SD)
-2.678	(0.033)	-6.086	(0.034)
-7.905	(0.030)	2.172	(0.033)
2.806	(0.073)	-0.450	(0.080)
-1.151	(0.261)	0.047	(0.300)
-0.702	(0.210)	0.029	(0.252)

P1-Cu^{2+}

F_H_2O	(SD)	ΔF_^2H_2O[a]	(SD)
-2.365	(0.011)	-6.071	(0.013)
-7.024	(0.011)	2.022	(0.015)
2.511	(0.022)	-0.251	(0.029)
-0.793	(0.073)	0.006	(0.116)
-0.771	(0.085)	0.006	(0.121)

P3

F_H_2O	(SD)	$\Delta F\_{}^2H_2O$[a]	(SD)
-4.066	(0.154)	-6.014	(0.411)
-9.218	(0.348)	2.449	(0.432)
3.969	(0.150)	-0.435	(0.202)
-0.566	(0.140)	0.002	(0.173)
-1.227	(0.079)	0.005	(0.102)

P3-Cu^{2+}

F_H_2O	(SD)	$\Delta F\_{}^2H_2O$[a]	(SD)
-1.722	(0.057)	-5.497	(0.244)
-7.234	(0.231)	1.700	(0.294)
1.550	(0.062)	-0.256	(0.082)
-1.074	(0.081)	0.032	(0.103)
-0.943	(0.228)	0.028	(0.260)

[a] $\Delta f\_{}^2H_2O$ refers to the difference structure factors between a sample measured in H_2O and the same sample measured in 80:20 H_2O:^2H_2O.

Table S3. Neutron Diffraction (ND): Structure factors and standard deviations (SD) from ND experiments done on POPC aligned multilayers in the presence of deuterated P3-Cu^{2+}.

F_H_2O	(SD)	$\Delta F\_{}^2H_2O$	(SD)	ΔF_D15[a]	(SD)	ΔF_D30[b]	(SD)
-1.075	(0.022)	-4.929	(0.028)	-0.477	(0.024)	-0.056	(0.024)
-5.246	(0.017)	1.047	(0.032)	-0.418	(0.019)	-0.382	(0.019)
1.168	(0.065)	-0.124	(0.121)	0.330	(0.069)	-0.126	(0.073)
-0.716	(0.077)	0.053	(0.213)	0.014	(0.104)	0.100	(0.139)
-0.554	(0.219)	0.041	(0.314)	-0.022	(0.256)	-0.014	(0.275)

[a]Δf_d15 is the difference in structure factors between a sample containing P3 deuterated in the N-terminal region ($I_5^{d10}F_6^{d5}$:15 deuterons) and unlabeled P3.

[b]Δf_d30 is the difference structure factors between a sample containing P3 deuterated in the N- and C-terminal regions ($I_5^{d10}F_6^{d5}$- $F_{19}^{d5}L_{20}^{d10}$: 30 deuterons) and unlabeled P3.

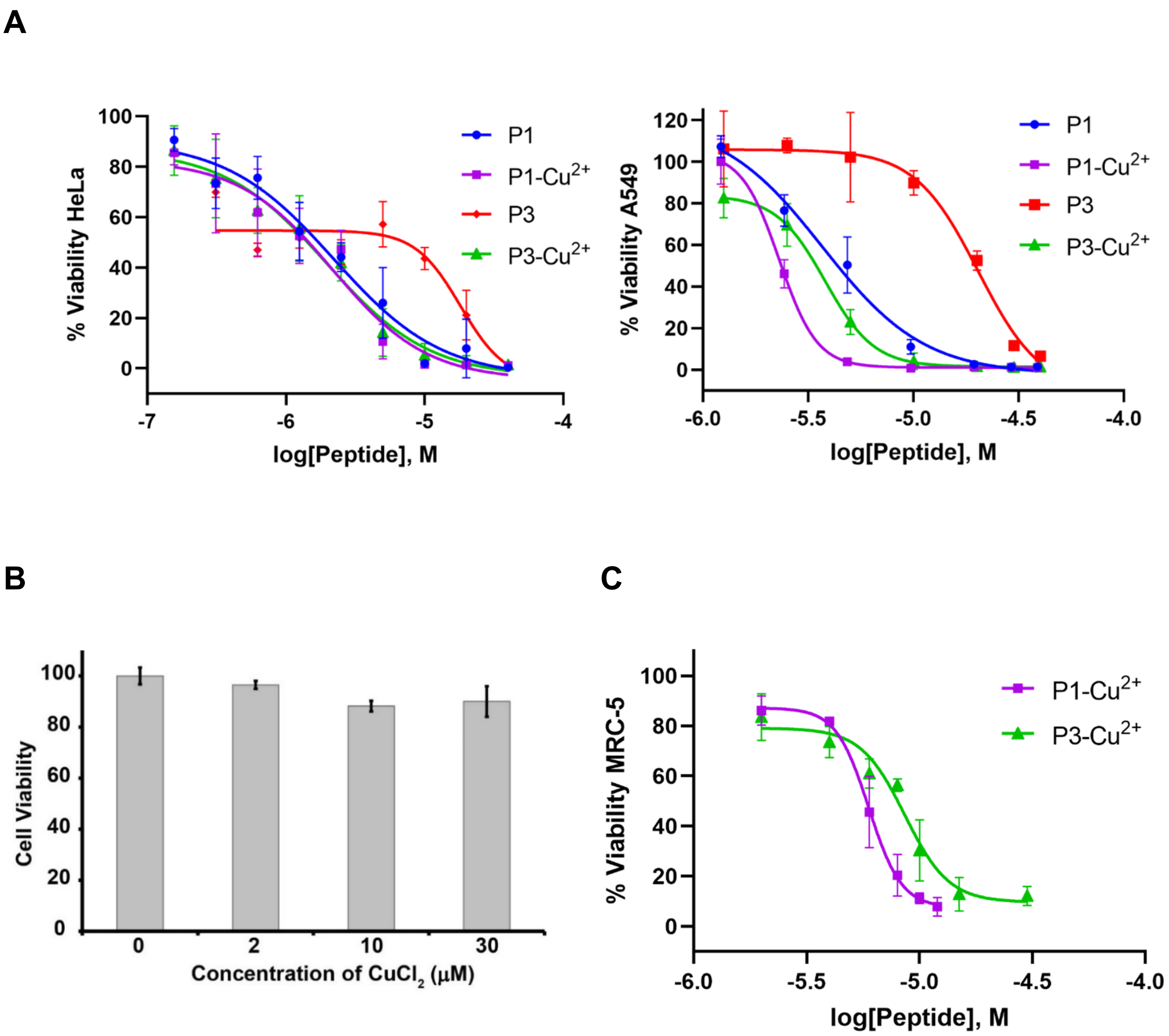

Figure S1. Cytotoxicity assays for P1 and P3 acting in the apo- and holo-states, and control experiments with $CuCl_2$ and MRC-5 cells. (A) The peptides were tested on HeLa cells as tabulated in Table 1. The graphed data are displayed here (left). As described in Figure 1, the lung cancer cell line (A549) was used to measure the cell viability upon treatment with P1 and P3 in the metal-free (apo) and Cu^{2+}-bound (holo). The results after 48 h of treatment are presented here (right). (B) Cu^{2+} (as an aqueous solution of $CuCl_2$) was added to the HT1080 cells in the free-state instead of being coordinated to the piscidin. No cell death was achieved by free Cu^{2+} even though it is a strong oxidant, possibly because it would have been precipitated by the DPBS. That Cu^{2+} in the free state does not kill the

cells but it does bound to piscidin suggest that it does not reach the cancer cells unless it is bound to piscidin. (C) P1-Cu^{2+} and P3-Cu^{2+} were assayed on normal MRC-5 (lung) fibroblast cells (ATTC catalog no CCL-171), giving rise to respective IC_{50} values of 5.97 (5.61-6.34) and 8.62 (7.56-9.82) μmol/L, with the 95% confidence interval given in parentheses. Apo-P1 was also tested as a control, giving rise to 50% inhibition at 8.56 μmol/L, in good agreement with the work of Lin et al.[1] where apo-P1 resulted in ~45% inhibition at about 10 μmol/L (27.5 μg/mL). Tested at up to 10 μ mol/L on other normal fibroblast cell lines, including HGF-1 (gingival) and OMF (mucosal), apo-P1 exhibited no significant inhibitory effects (survival > 90%) based on a recent study by Cheng et al.[2]. Thus, among normal cells tested with apo-P1, the MRC-5 cells appear to be the most sensitive.

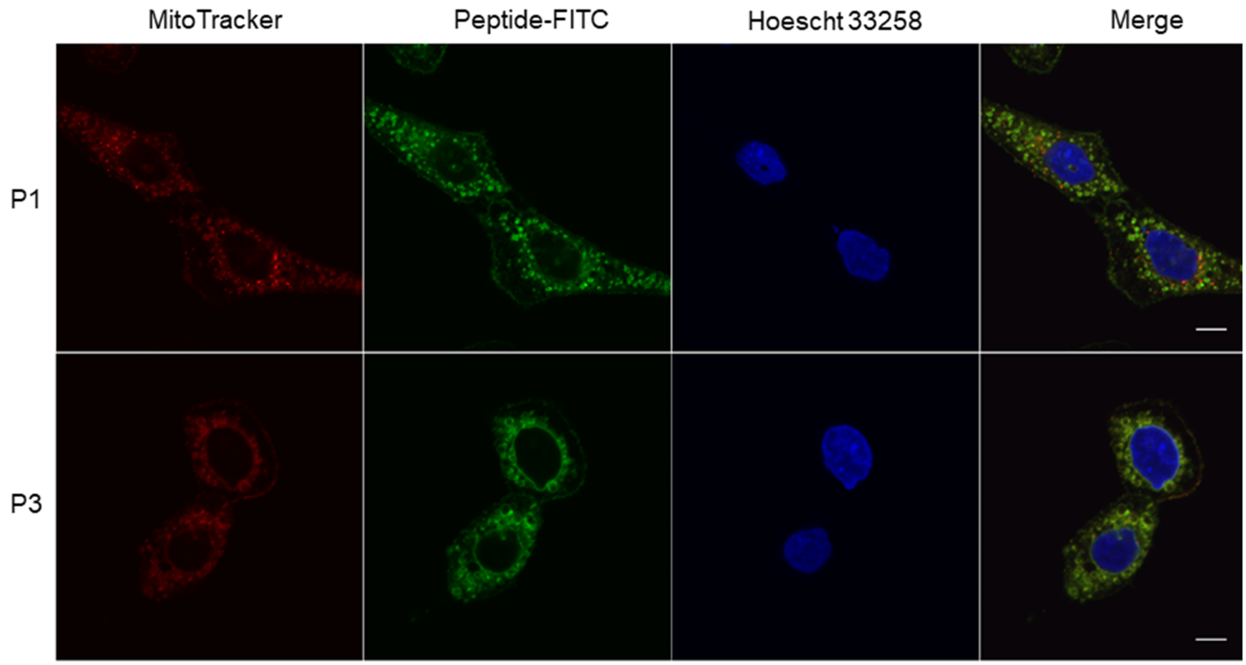

Figure S2. HeLa cells incubated with FITC-labeled P1 and P3. HeLa cells were exposed to 100 nmol/L MitoTracker (red), 5 µg/mL Hoescht 33258 (DNA stain), and 4 µM FITC-labeled peptides (green) for 20 mins. Top row: FITC-P1 stains the cellular membrane and nuclear envelope and co-localizes with the MitoTracker. Bottom row: FITC-P3 stains the cellular membrane and there is also some co-localization with MitoTracker (left cell). The scale bar represents 5 µm.

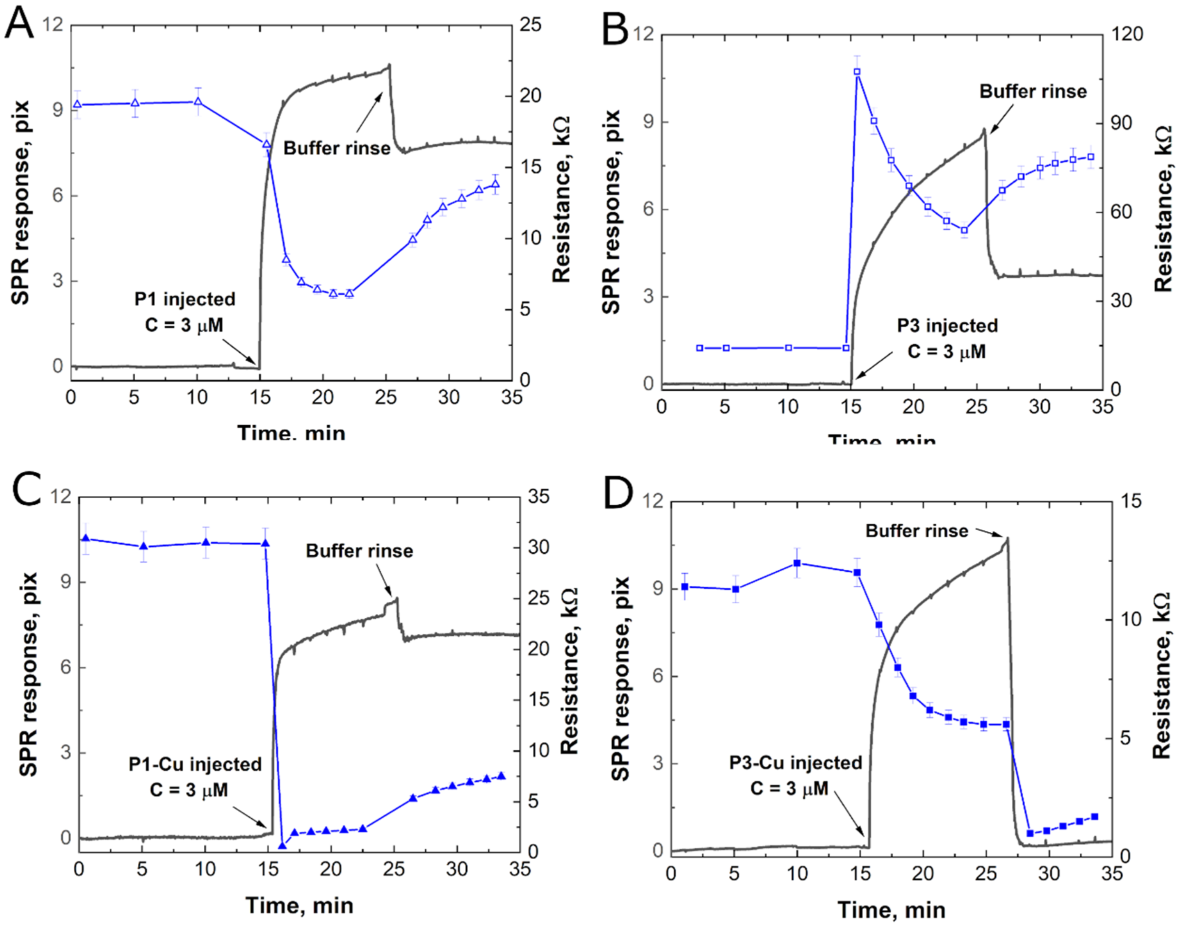

Figure S3. SPR/EIS data at a peptide concentration of 3 μmol/L. The SPR/EIS signals were monitored simultaneously on the tBLM as a function of time, starting after the establishment of a stable tBLM: (A) P1, (B) P3, (C) P1-Cu^{2+}, and (D) P3-Cu^{2+}. The black curves are the SPR responses, and the blue curves are the bilayer resistance determined from the EIS measurements. While binding kinetics (SPR signals) are almost similar for the apo- and holo-states, the peptides differ in their ability to form defects in the bilayers (EIS signals). P1 deform the membrane instantly after its addition whereas P3 first adsorbs onto tBLM. Metal-binding clearly enhances the formation of the bilayer defects by both peptides, as evidence by the irreversible decrease in resistance. The impact of metallation on P3 is very pronounced. As indicated by the drop in SPR signal upon buffer, the metallated peptide seems to lead to the removal of POPC molecules from the supported bilayer.

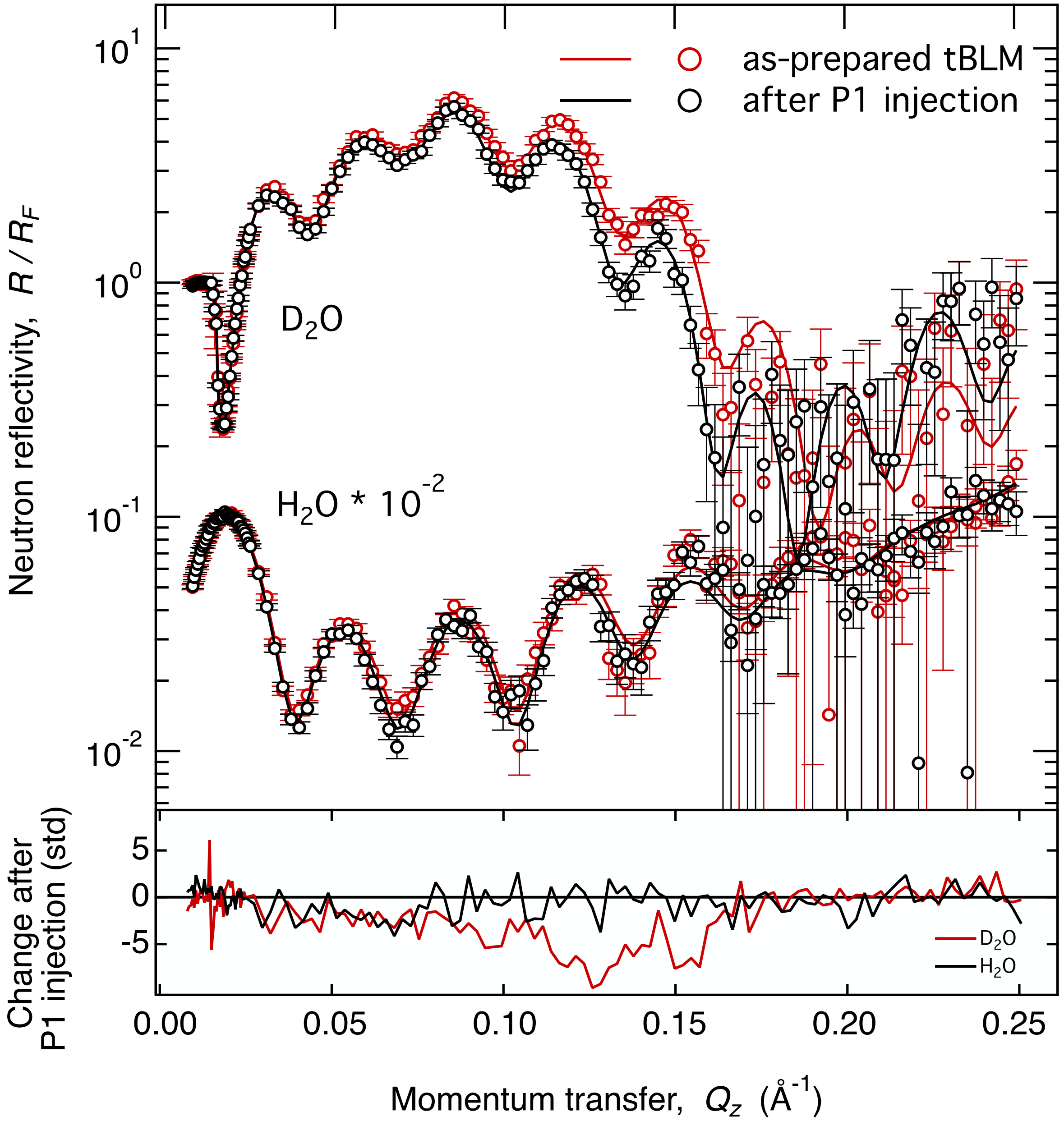

Figure S4. Representative neutron reflectivity (NR) curves. Representative Fresnel-normalized NR curves are shown for POPC tBLM before and after exposure to 3 μmol/L P1. Error bars signify one standard deviation. The inset at the bottom of the graph shows the changes in reflectivity after adding P1 (error-weighted residuals between reflectivities obtained before and after adding P1). Qualitatively similar reflectivity curves were recorded for P1-Cu^{2+}, P3, and P3-Cu^{2+}.

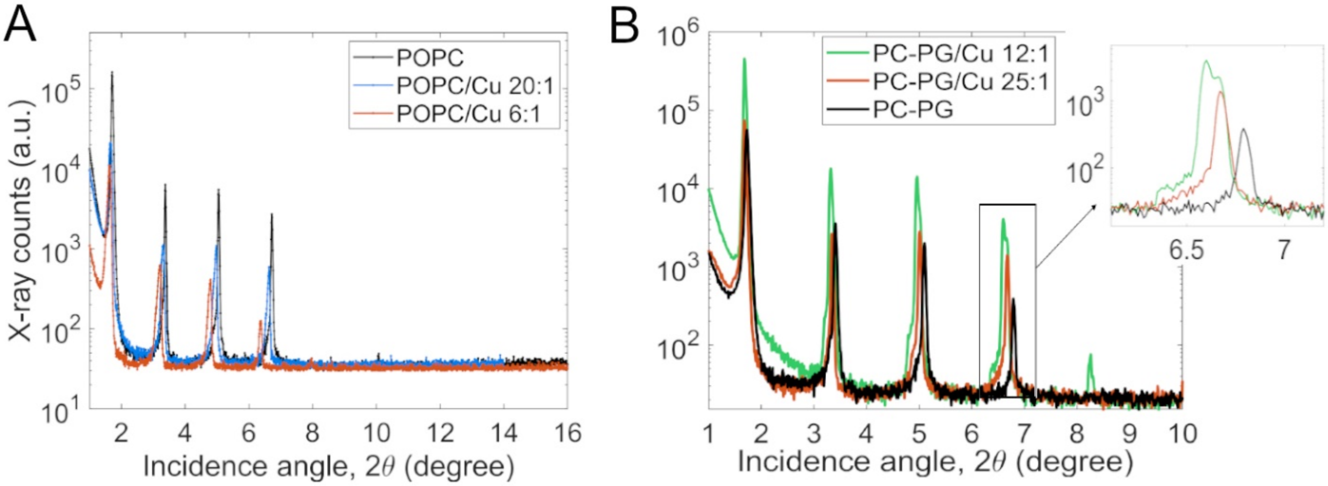

Figure S5. Representative X-ray diffraction data for lipid multilayers in the presence of Cu^{2+}. (A) $CuCl_2$ was co-solubilized with POPC lipid in 2 mM phosphate buffer at various POPC-to-Cu^{2+} molar ratios. Multilayers were formed by fusing the liposomes on a solid substrate and letting them spread and orient. Lamellar diffraction was collected after each sample was equilibrated for 24 hours in a humid atmosphere at 98% relative humidity and 25 °C. Repeat spacings are: 52.9 ± 0.02 Å (POPC), 53.4 ± 0.1 Å (POPC/Cu^{2+} 20:1) and 55.9 ± 0.1 Å (POPC/Cu^{2+} 6:1). The increase in the repeat with Cu^{2+} is likely due to the presence of the metal ions in the headgroups[3]. (B) Samples were made of POPC/POPG 3:1 with small amounts (10% molar) of 1-palmitoyl-2-(9'-oxo-nonanoyl)-sn-glycero-3-phosphocholine (AldoPC) that does not affect the diffraction data compared to POPC/POPG (data not shown). Samples with $CuCl_2$ were prepared as in (A). Inset shows a zoom-in on the 4th diffraction peak, showing signs of phase separation (peak splitting) at the 12:1 lipid/Cu^{2+} ratio, possibly due to preferential binding of Cu^{2+} to the negatively charged PG headgroups. Repeat spacings are: 52.2 ± 0.1 Å (PC/PG), 53.1 ± 0.03 Å (PCPG/Cu^{2+} 25:1), and 53.9 ± 0.1 Å (PCPG/Cu^{2+} 12:1).

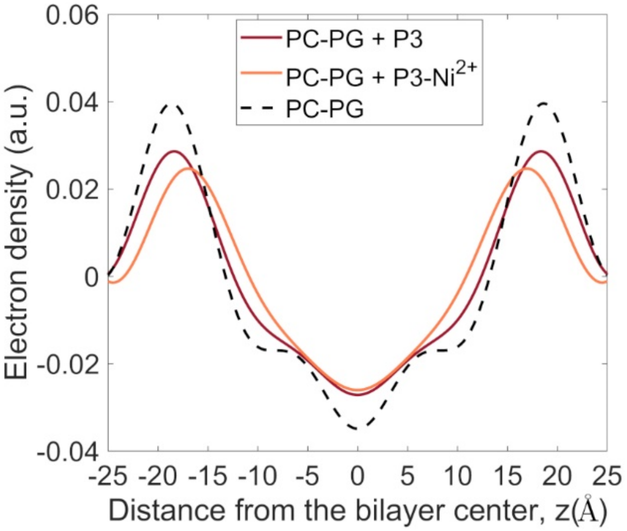

Figure S6. Electron density profiles for P3-Ni^{2+} in bilayers. P3 in the apo- (orange) or Ni^{2+}-bound (dark red) states were incorporated in multilayers comprised of POPC/POPG 3:1, with 10% Aldo-PC (See Figure S5). X-ray diffraction data were collected and analyzed, as described previously[4]. The peptide-to-lipid molar ratio was 1:50. Measurements were done at 98% relative humidity and 25 ºC. Profiles show a density redistribution and migration toward the bilayer center when Ni^{2+} is present. The profile of the lipid without peptide is shown in black. Corresponding repeat distances are: 52.2 ± 0.1 Å (lipid, black), 51.5 ± 0.2 Å (lipid + P3, dark red), and 49.0 ± 0.1 Å (lipid + P3-Ni^{2+}, orange).

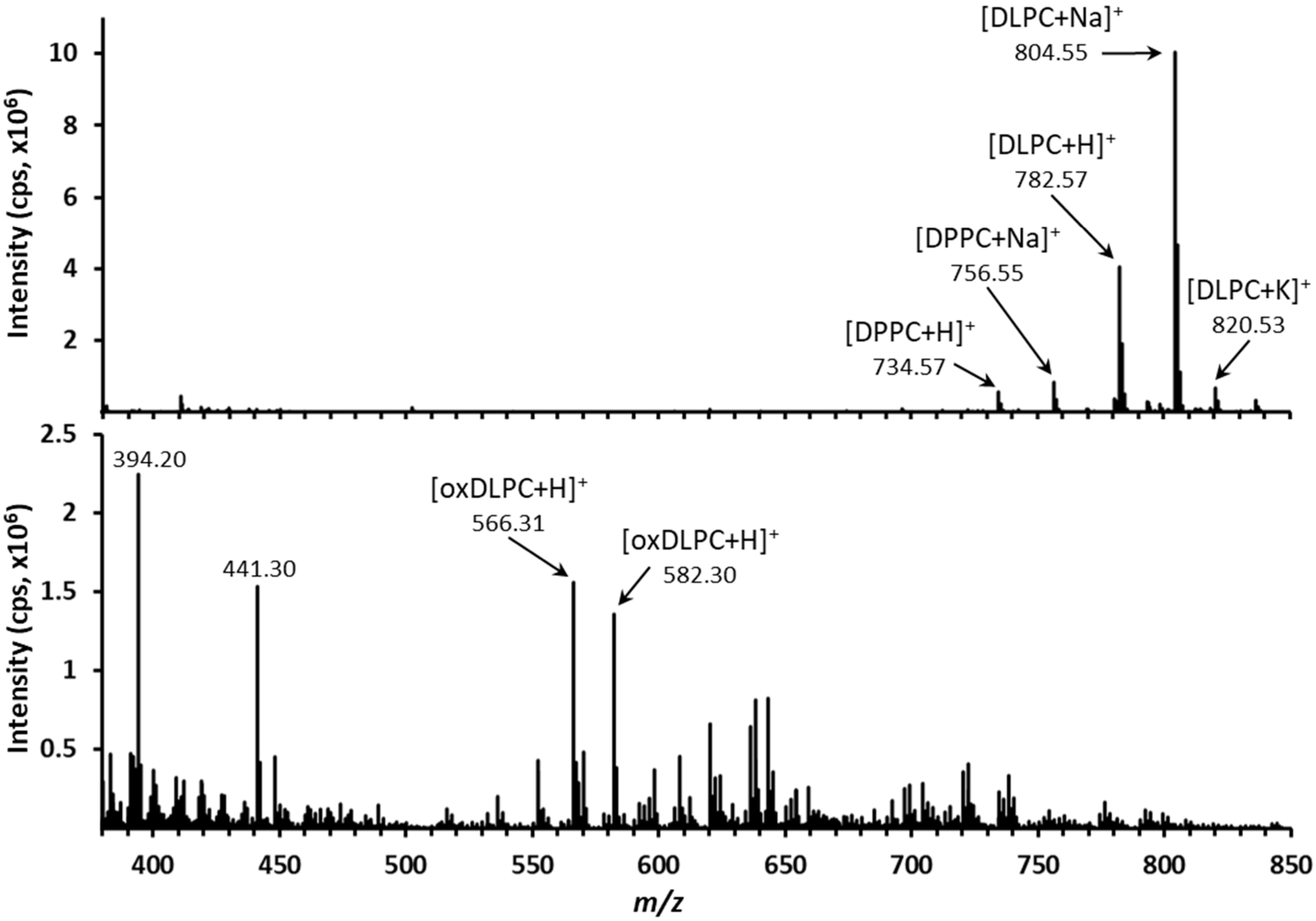

Figure S7. **Direct infusion electrospray mass spectrometry analysis of oxidation products formed from exposing DLPC to Cu^{2+} ions.** Top: DLPC. Bottom: DLPC/Cu^{2+} (2:1). The samples were prepared as liposomes dispersed in 1:1 H_2O:methanol, starting with freshly open DLPC. The spectra were collected 20 h after exposure. The peak at m/z = 566 is DLPC with both chains truncated and terminated with -CHO, the peak at m/z = 582 is DLPC with both chains truncated with one terminated with –CHO and the other with –COOH.

REFERENCES

1 Lin, H.-J. *et al.* Piscidin-1, an Antimicrobial Peptide from Fish (Hybrid Striped Bass Morone saxatilis x M. chrysops), Induces Apoptotic and Necrotic Activity in HT1080 Cells. *Zoolog Sci* **29**, 327-332 (2012).

2 Cheng, M. H. *et al.* Piscidin-1 Induces Apoptosis via Mitochondrial Reactive Oxygen Species-Regulated Mitochondrial Dysfunction in Human Osteosarcoma Cells. *Sci Rep* **10**, 5045 (2020).

3 Poyton, M. F., Sendecki, A. M., Cong, X. & Cremer, P. S. Cu^{2+} Binds to Phosphatidylethanolamine and Increases Oxidation in Lipid Membranes. *J Am Chem Soc* **138**, 1584-1590 (2016).

4 Comert, F. *et al.* The host-defense peptide piscidin P1 reorganizes lipid domains in membranes and decreases activation energies in mechanosensitive ion channels. *J Biol Chem* **294**, 18557-18570 (2019).
